# A model of on/off transitions in neurons of the deep cerebellar nuclei: deciphering the underlying ionic mechanisms

**DOI:** 10.1186/s13408-021-00105-3

**Published:** 2021-04-01

**Authors:** Hugues Berry, Stéphane Genet

**Affiliations:** 1 INRIA, 69603 Villeurbanne, France; 2 LIRIS UMR5205, Université de Lyon, 69622 Villeurbanne, France; 3 Sorbonne Université, CNRS, Institut des Systèmes Intelligents et de Robotique, ISIR, F-75005 Paris, France

**Keywords:** Deep cerebellar nuclei, State transition, Biophysical model

## Abstract

The neurons of the deep cerebellar nuclei (DCNn) represent the main functional link between the cerebellar cortex and the rest of the central nervous system. Therefore, understanding the electrophysiological properties of DCNn is of fundamental importance to understand the overall functioning of the cerebellum. Experimental data suggest that DCNn can reversibly switch between two states: the firing of spikes (F state) and a stable depolarized state (SD state). We introduce a new biophysical model of the DCNn membrane electro-responsiveness to investigate how the interplay between the documented conductances identified in DCNn give rise to these states. In the model, the F state emerges as an isola of limit cycles, i.e. a closed loop of periodic solutions disconnected from the branch of SD fixed points. This bifurcation structure endows the model with the ability to reproduce the $\text{F}\to \text{SD}$ transition triggered by hyperpolarizing current pulses. The model also reproduces the $\text{F}\to \text{SD}$ transition induced by blocking Ca currents and ascribes this transition to the blocking of the high-threshold Ca current. The model suggests that intracellular current injections can trigger fully reversible $\text{F}\leftrightarrow \text{SD}$ transitions. Investigation of low-dimension reduced models suggests that the voltage-dependent Na current is prominent for these dynamical features. Finally, simulations of the model suggest that physiological synaptic inputs may trigger $\text{F}\leftrightarrow \text{SD}$ transitions. These transitions could explain the puzzling observation of positively correlated activities of connected Purkinje cells and DCNn despite the former inhibit the latter.

## Introduction

The connectivity of the cerebellum places deep cerebellar nuclei neurons (DCNn) in a strategic position. Their axons are the main output of the cerebellum, projecting to the forebrain, the brain stem and the spinal cord. In turn, DCNn are the main target of the Purkinje cell (PC) axons, which are themselves the sole output system of the cerebellar cortex. As noted by Lllinás and Muhlethaler [1] the cerebella nuclear cells are therefore the main functional link between the cerebellar cortex and the rest of the central nervous system. It follows that a detailed understanding of electrophysiological properties of the DCNn is one of the fundamental prerequisites to understanding the overall functioning of the cerebellum. Several experimental studies have demonstrated that DCNn are not simple leaky integrators of their synaptic inputs but exhibit a repertoire of intrinsic active electric properties. Recordings using sharp electrodes or patch clamps have evidenced that isolated DCNn are spontaneously active, producing pacemaker firing of fast spikes at a low frequency of about 20 Hz [2–7]. DCNn also exhibit rebound depolarizing potentials at break of a hyperpolarizing current pulse in a variety of preparations (see [3]). In addition, Jahnsen [2] reported that pulses of injected hyperpolarizing current can switch DCNn from their spontaneous firing state to a silent mode of activity. As this silent mode was observed to persist over time spans ${>}1~\mbox{s}$, Jahnsen’s results putatively point at the existence of a stable point attractor in the DCNn phase space. Moreover, the observation of Jahnsen that a subsequent hyperpolarizing pulse can switch the DCNn back to their spontaneous firing activity (see Fig. 5 in [2]) suggests that transitions between the two modes of activity are reversible. Reversible transitions between spontaneous firing and a silent depolarized state were also reported later by Raman *et al*. [4] in patch-clamp recordings of DCNn, thus ruling out the possibility that these transitions are artifacts resulting from experimental membrane leaks created by sharp electrodes used by Jahnsen. While sparse, these experimental results hint at the coexistence of two different stable electric states in the DCNn phase space: a silent depolarized state (stable fixed point, sFP) and an active state characterized by low frequency firing of large-amplitude spikes (stable limit cycle, sLC).

Such dynamics was early predicted as possible solutions of the Hodgkin–Huxley (HH) model of the action potential by Cooley *et al*. as early as 1965 [8]. They pointed out that (i) if the HH model had only two variables, the surrounding of the sFP by the orbit of the sLC (see Theorem 6.2 in Grimshaw [9]) would imply the existence of an unstable limit cycle (uLC) to separate the basins of attraction of the two *ω*-limit sets but (ii) that the existence of such an uLC is not a necessary condition for the (four-dimensional) HH model to exhibit such dynamics. Hassard and Shiau [10] and Rinzel and Miller [11] subsequently showed that the HH model has actually a branch of unstable limit cycles in addition to the branch of limit cycles in the range of input currents where the sFP and the sFP coexist. Moreover Guttman *et al*. [12] subsequently proved the physiological soundness of the premises of Cooley et al. [8] by reporting that the squid axon can be switched by short pulses of electric current between a silent mode and a firing mode. They also went beyond Cooley *et al*. [8] by suggesting that, since the HH model has a four-dimensional phase space, the basins of attraction of the sFP and the sLC are separated by a $d \ge 2$ manifold rather than by a single curve and that this separatrix presumably contains the previously identified uLC.

These conjectures deserve explanation. Firstly, uniqueness of the solution of a Cauchy problem for systems of ordinary differential equations (ODE) imply that, in a 2D phase space, the separatrix between a sLC and a sFP must be a Jordan curve, say *C*, obviously corresponding to a limit cycle solution of the system. Secondly, this limit cycle must be unstable to ensure that all solutions starting from the interior (*resp*. exterior) of *C* converge onto the sFP (*resp*. the sLC). A limit cycle always has one of its Floquet exponents $\rho _{1} = 1$ (see e.g. [13], p. 223) and, for a 2D system, its second exponent $\rho _{2}$ is >1 if the limit cycle is unstable. The local stable and unstable manifolds of the uLC are, respectively, spanned by $\rho _{1}$ and $\rho _{2}$ and both have dimension 1. According to the stable manifold theorem for periodic orbits (see [13], p. 220), the global stable (*resp*. unstable) manifolds of the uLC have the same respective dimensions as the local stable (*resp*. unstable manifolds of the uLC. It is therefore tempting to conjecture that, in systems with dimension >2, the separatrix is also the global stable manifold of the uLC, that is a manifold with dimension $k + 1$ with *k* being the number of Floquet exponents <1. Such a manifold may have a complicated shape. But if solutions are bounded, the simplest manifold achieving separation of the attraction basins of the sFP and sLC would be a closed manifold (i.e. compact and without bounds). Characterizing the separatrix may have remained a theoretical issue since the coexistence of the sFP and the sLC has, to our knowledge, only be reported in squid axons bathed in non-physiological low Ca salines. However, the observation of such a coexistence in DCNn put this mathematical question into a neurophysiological context since characteristics of the separatrix obviously constrain the types of stimuli capable to trigger $\mathrm{sFP} \leftrightarrow \mathrm{sLC}$ state changes.

The repertoire of membrane conductances expressed by DCNn has been the subject of several studies. These conductances comprise two calcium carried currents [14], a non-inactivating high-threshold Ca current ($I_{\mathrm{CaH}}$) and an inactivating (transient) one ($I_{\mathrm{CaT}}$) and a calcium-dependent potassium current ($I_{\mathrm{KCa}}$, [15]). In addition, Raman *et al*. [4] have identified a tonic non-cationic current, $I_{\mathrm{TCN}}$. However, in spite of these characterizations, we still lack an understanding of how this set of conductances actually implements the intrinsic properties of the DCNn. In the absence of a theoretical framework, it is difficult to relate the response of the DCNn membrane voltage to its active conductances. In particular, several of the available experimental observations raise unsolved issues. For instance, transitions from the spontaneous firing state to the silent depolarized one can be triggered by inhibition of the calcium currents $I_{\mathrm{CaH}}$ and $I_{\mathrm{CaT}}$ [4] but it is not clear whether the inhibition must concern both currents or only one of them. Likewise, it is not known if the transitions observed *in vitro* using current pulse injection are also triggered by synaptic currents.

Biophysical models have proven highly useful to understand how active conductances interact to underpin active electrical signals in neurons. For instance, the acclaimed Hodgkin and Huxley model of the action potential in the squid axon has proved a very powerful approach to our understanding of the generation of action potentials [16]. For DCNn, the only detailed (compartmental) biophysical model of DCNn thus far (to our knowledge) has been proposed in a pioneer study by Steuber *et al*. [7]. The more recent model of Sudhakar *et al*. [17] actually adopts the structure and equations of the Steuber *et al*.’s model and only uses different values for the densities of active conductances. However, neither of these articles evidences capabilities of this model to reproduce state transitions experimentally observed by Jahnsen [2] and Raman *et al*. [4]. Moreover, the Steuber *et al*.’s model relies on several assumptions which experimental support can be questioned. Thus, it assumes that DCNn express two different Hodgkin–Huxley-type voltage-dependent Na currents, a fast one and a slowly inactivating one (i.e. persistent). Expression of a persistent Na current by DCNn was postulated by Jahnsen [2] and Lllinás and Muhlethaler [1] and its existence has since been confirmed by two experimental studies (Raman et al. [4] and Afshari et al. [18]). However, these studies suggest that the persistent Na current belongs to the class of so-called resurgent Na currents (Raman and Bean, [19]). Resurgent currents involve a specific mode of channel aperture whose faithful modeling requires a set of 12 ODEs (Raman and Bean, [19]). Moreover, the Steuber *et al*.’s model assumes a non-uniform distribution of active conductances between the dendrites and soma of DCNn which remained to be proved (with the exception of T Ca channels that cluster to the soma and proximal dendrites of DCNn; see McKay et al. [20]). Here we propose a new biophysical model of DCNn electrogenesis. To allow mathematical analysis of the model, we focus on an isopotential (non-compartmental) description, in which the DCNn membrane potential evolves under the influence of the 6 conductances mentioned above ($I_{\mathrm{NaV}}$, $I_{\mathrm{Kdr}}$, $I_{\mathrm{CaH}}$, $I_{\mathrm{CaT}}$, $I_{\mathrm{KCa}}$, and $I_{\mathrm{TCN}}$). Spontaneous firing of fast spikes emerges in the model as an isola of limit cycles in a range of driving currents. Within this range, the model exhibits another coexisting stable attractor: a stable depolarized stationary-state. Owing to this peculiar bifurcation structure, the model reproduces the transitions between spontaneous firing and a silent state that have been reported experimentally [2, 4]. Like in these experimental reports, the transitions in the model can be triggered by hyperpolarizing current pulses or by blocking Ca currents in the absence of hyperpolarization. Analysis of the model suggests that the latter transition is specifically due to the inhibition of the $I_{\mathrm{CaH}}$ current. Finally, simulations of the model suggest that physiological synaptic inputs may trigger the state transitions.

## Methods

It is currently unknown whether DCNn are electrically compact neurons nor whether their ion channels have non-uniform distributions over their membrane surface (with the exception of high threshold voltage-dependent Ca channels [14]). In the absence of evidence for a functional role of these heterogeneities, we chose to build an isopotential model of DCNn to (i) investigate the hypothesis that DCNn have two coexisting stable states of activity, (ii) identify the interactions between the active membrane ion currents that are responsible for this electric feature and (iii) investigate whether physiological inputs could trigger transitions between these states. DCNn do not form a homogeneous population from the phenotypic standpoint. Their population has been divided into two classes (see [21] for an introduction) which partially correlate with the location of their target neurons. Glutamatergic DCNn project excitatory inputs to motor centers in the brain stem, the mesencephalon, the thalamus and pre-cerebellar nuclei though the mossy fiber pathway. GABAergic DCNn achieve inhibitory synapses on neurons of the inferior olive [22] and glutamatergic DCNn [23]. A class of rare small-sized DCNn has also been identified [24]. In addition to their neurotransmitters and projection sites, these classes of neurons can also express different sets of ion channels [25]. However, it is unknown to what extent their intrinsic electrophysiological properties are heterogeneous. In the absence of this information, we propose below a generic model for the electrogenesis of DCNn.

### State variables and equations of the model

Our standard model has 6 state variables. The dynamics of these variables is governed by 6 associated ordinary differential equations (ODE) that we describe below. The two main state variables of the model are the membrane potential, *V* (mV), and the cytoplasmic concentration of free $\mathrm{Ca}^{2+}$ ions $[ \mathrm{Ca} ]$ (*μ*M). Their respective ODE read

1 $$ C\frac{dV}{dt} = - \sum_{i} I_{i}^{\mathrm{ion}} + I_{S} $$

and

2 $$ \frac{d [ \mathrm{Ca} ]}{dt} = - \frac{10^{2} ( I_{\mathrm{CaH}} + I_{\mathrm{CaT}} )R_{c}/F + 2k ( [ \mathrm{Ca} ] - [ \mathrm{Ca} ]_{b} ) ( R_{c} - \delta )}{\delta ( 2R_{c} - \delta ) [ 1 + \frac{B_{T}}{K_{D} ( 1 + \frac{10^{ - 11} [ \mathrm{Ca} ]}{K_{D}} )^{2}} ]}. $$

Equation (1) is standard for biophysical models of neurons (see e.g. [26]). It can be derived from the first Kirchhoff law of electromagnetism which states that electric charges can neither be created nor destroyed. The set of membrane ion currents $I_{i}^{\mathrm{ion}}$ (nAcm^−2^) appearing in Eq. (1) is detailed below. For the input current $I_{S}$ we distinguish tonic ($I_{\mathrm{DC}}$) and phasic ($I_{\varphi }$) components and write $I_{S} = I_{\mathrm{DC}} + I_{\varphi } $. The phasic component was written as the product of an ohmic term $g_{\varphi } ( V - E_{\varphi } )$ and the product of two Heaviside step functions of time $H ( t - t_{\mathrm{start}} )H ( t_{\mathrm{end}} - t ) = \big\{ \scriptsize{\begin{array}{l@{\quad}l} 1& \text{for } \mathrm{start} < t < \mathrm{end}, \\ 0& \text{elsewhere}. \end{array}}$ The ODE for the cytoplasmic free calcium ions concentration (Eq. (2)) is a balance equation. The source term corresponds to calcium ions entry into the cytoplasm through membrane channels underlying the $I_{\mathrm{CaH}}$ and $I_{\mathrm{CaT}}$ currents. The sink term corresponds to the extrusion of cytoplasmic Ca^2+^ ions by cytoplasmic membrane pumps and their internalization by Ca pumps in the endoplasmic reticulum membrane, both processes being modeled with a simple linear term. The ODE actually includes a second sink term corresponding to Ca^2+^ ions buffering by Ca-binding proteins. It appears as the denominator of the right-hand side of Eq. (2) owing to the hypothesis that the binding of Ca^2+^ ions is very fast (see [27] for the derivation of Eq. (2) and Table 1 for parameter values).

**Table 1:** List of parameter values. The abbreviation ‘cstd’ indicates that the value was constrained to reproduce experimental dynamics of DCN

Parameter	Value	Reference
*Calcium ions dynamics*		
$R_{c}$	$5 \times 10^{ - 4}~\mbox{cm}$	[26]
*δ*	$3 \times 10^{ - 4}~\mbox{cm}$	
*k*	$10^{ - 2}~\mbox{cm/s}$	
$[ B ]_{T}$	$1.5 \times 10^{2}~\mu \mbox{M}$	
$K_{D}$	$1~\mu \mbox{M}$	
*Membrane potential dynamics*		
*C*	$1~\mu \mbox{F/cm}^{2}$	[25]
*Membrane ion currents*		
*Maximum conductances and permeabilities*		
$g_{\text{L}}$	$2 \times 10^{1}~\mu \mbox{S/cm}^{2}$	cstd
$g_{\text{NaV}}$	$5 \times 10^{3}~\mu \mbox{S/cm}^{2}$	
$g_{\text{Kdr}}$	$4.5 \times 10^{3}~\mu \mbox{S/cm}^{2}$	
$g_{\text{TCN}}$	$4.5 \times 10^{1}~\mu \mbox{S/cm}^{2}$	
$g_{\text{KCa}}$	$10~\mu \mbox{S/cm}^{2}$	
$P_{\text{CaH}}$	$2 \times 10^{ - 4}~\mbox{cm/s}$	
$P_{\text{CaT}}$	$7 \times 10^{ - 4}~\mbox{cm/s}$	
*Nernst potential*		
$E_{\text{L}}$	$- 60~\mbox{mV}$	cstd
$E_{\text{Na}}$	$+ 86~\mbox{mV}$	[4]
$E_{\text{K}}$	$- 80~\mbox{mV}$	$-83~\mbox{mV}$ in [28]
$E_{\text{Cl}}$	$- 75~\mbox{mV}$	$-75~\mbox{mV}$ [2] and $-74.3~\mbox{mv}$ in [28]; however, see [5]
$E_{\text{TCN}}$	$- 34~\mbox{mV}$	[4]
*Steady-state (in)activation parameters (mV)*		
$V_{m_{\text{NaV}}}$	−32	cstd
$k_{m_{\text{NaV}}}$	8.5	
$V_{h_{\text{NaV}}}$	−55	
$k_{h_{\text{NaV}}}$	5.5	[4]
$V_{m_{\text{Kdr}}}$	−25	[29]
$k_{m_{\text{Kdr}}}$	11.5	
$V_{m_{\text{CaH}}}$	−22	cstd
$k_{m_{\text{CaH}}}$	4.53	
$V_{m_{\text{CaT}}}$	−56	[30]
$k_{m_{\text{CaT}}}$	6.2	
$V_{h_{\text{CaT}}}$	−80	
$k_{h_{\text{CaT}}}$	4	
*Parameters of time constants*		
$\tau _{n_{\text{Kdr}}0}$	$0~\mbox{s}$	cstd
$\tau _{n_{\text{Kdr}}1}$	$5.4 \times 10^{ - 3}~\mbox{s}$	
$\alpha _{\tau _{n_{\text{Kdr}}}}$	6 × 10^−1^	
$V_{\tau_{n_{\text{Kdr}}1}} = V_{\tau_{n_{\text{Kdr}}2}}$	$- 30~\mbox{mV}$	
$k_{\tau_{n_{\text{Kdr}}1}} = k_{\tau_{n_{\text{Kdr}}2}}$	$25~\mbox{mV}$	
$\tau _{h_{\text{NaV}}0}$	$5 \times 10^{ - 3}~\mbox{s}$	
$\tau _{h_{\text{NaV}}1}$	$3 \times 10^{ - 2}~\mbox{s}$	
$\alpha _{\tau _{h_{\text{NaV}}}}$	1	
$V_{\tau_{h_{\text{NaV}}1}} = V_{\tau_{h_{\text{NaV}}2}}$	$- 65~\mbox{mV}$	
$k_{\tau_{h_{\text{NaV}}1}} = k_{\tau_{h_{\text{NaV}}2}}$	$7~\mbox{mV}$	
$\tau _{m_{\text{CaT}}0}$	$2 \times 10^{ - 4}~\mbox{s}$	[30]
$\tau _{m_{\text{CaT}}1}$	3.33 × 10^−4^	
$V_{\tau_{m_{\text{CaT}}1}}$	$- 131~\mbox{mV}$	
$k_{\tau_{m_{\text{CaT}}1}}$	$16.7~\mbox{mV}$	
$\alpha _{\tau _{m\text{CaT}}}$	1	
$V_{\tau_{m_{\text{CaT}}2}}$	$- 15.8~\mbox{mV}$	
$k_{\tau_{m_{\text{CaT}}2}}$	$18.2~\mbox{mV}$	
$\tau _{h_{\mathrm{CaH}}0}$	$1.2 \times 10^{ - 2}~\mbox{s}$	cstd (to produce a $C^{1}$ function for $\tau _{h_{\mathrm{CaT}}}$)
$\tau _{h_{\mathrm{CaH}}1}$	$2~\mbox{s}$	
$\alpha _{\tau _{h_{\mathrm{CaT}}}}$	1	
$V_{\tau_{h_{\mathrm{CaH}}1}} = V_{\tau_{h_{\mathrm{CaH}}2}}$	$- 81~\mbox{mV}$	
$k_{\tau_{h_{\mathrm{CaH}}1}} = k_{\tau_{h_{\mathrm{CaH}}2}}$	$8~\mbox{mV}$	

The ion membrane conductances (for currents obeying the Nernst equation) and permeabilities (for currents obeying the Goldman constant-field equation) were modeled as the product of a maximum conductance *g̅* (*μ*S/cm^2^) or permeability *P̅* (cm/s) and either the product of voltage-dependent activation and inactivation variables *m* and *h* or a Ca-dependent variable *w*, i.e.

3 $$ \begin{aligned} &g ( P ) = \overline{g} ( \overline{P} )m^{p}h^{q} , \\ &g ( \overline{P} ) = \overline{g} ( \overline{P} )w, \end{aligned} $$

in which *p* and *q* are integers. The remaining four states variables of the model correspond to in(activation) variables of ion currents $I_{\mathrm{NaV}}$, $I_{\mathrm{Kdr}}$ and $I_{\mathrm{CaT}}$ and namely are $h_{\mathrm{NaV}}$, $m_{\mathrm{Kdr}}$, $m_{\mathrm{CaT}}$ and $h_{\mathrm{CaT}}$. Their dynamics obey first order differential equations of the form [16]

4 $$ \forall x \in \{ h_{\mathrm{NaV}},m_{\mathrm{Kdr}},m_{\mathrm{CaT}},h_{\mathrm{CaT}} \} \frac{dx}{dt} = \frac{x_{\infty } - x}{\tau _{x}}, $$

in which $x_{\infty}$ and $\tau_{x}$ stand for the steady-state value and the exponential time constant of variable *x*, respectively. The available experimental data on the voltage-dependence in DCNn [4, 18] were insufficient to constrain a fully detailed Hodgkin–Huxley-type model of these currents since they do not document the voltage dependence of rate functions governing state transitions of ion channels. We therefore adopted the modeling approach of Hughenard and McCormick [29] in which $x_{\infty}$ and $\tau_{x}$ are considered as independent functions of *V* (however, see the discussion) of the form

5 $$\begin{aligned}& x_{\infty } = \frac{1}{1 + e^{ \pm ( V - V_{x} )/k_{x}}}, \end{aligned}$$

6 $$\begin{aligned}& \tau _{x} = \tau _{x_{0}} + \frac{\tau _{x_{1}}}{e^{ \pm ( V - V_{\tau _{x1}} )/k_{\tau _{x1}}} + \frac{\alpha _{\tau _{x}}}{e^{ ( V - V_{\tau _{x2}} )/k_{\tau _{x2}}}}}. \end{aligned}$$

Equation (5) is a so-called Boltzmann function in which the ‘−’ and ‘+’ signs hold, respectively, for the inactivation ($h_{\mathrm{NaV}}$, $h_{\mathrm{CaT}}$) and the activation ($m_{\mathrm{Kdr}}$, $m_{\mathrm{CaT}}$) variables. $V_{x}$ and $k_{x}$ are referred to as half-activation potentials and activation slopes of the variable *x*, respectively. Equation (6) is standard for reproducing the bell-shape of the activation time constant of voltage-dependent currents [29]. The parameter $\tau_{x_{0}}$ accounts for the observation that $\lim_{V \to + \infty } \tau _{x} > 0$ for the inactivation variable of some particular currents (e.g. $I_{\mathrm{Na}}$ in the squid giant axon [11]. The $\alpha _{\tau _{x}}$ parameter allows one to introduce asymmetry in the shape of the time constant as observed for some currents (see e.g. [26], p. 48). We give below a detailed account of the model equation for each of the currents.

$I_{\mathrm{NaV}}$: As in most neurons studied so far, the spikes of the DCNn are blocked by tetrodotoxin (TTX) [4] suggesting that the depolarizing phase of these spikes is underlain by a Hodgkin–Huxley-type of voltage-dependent Na current, $I_{\mathrm{NaV}}$. For this reason, we used the classical Hodgkin–Huxley formalism to model $I_{\mathrm{NaV}}$ in DCNn. However, the time constant of $m_{\mathrm{NaV}}$ in neurons is usually much smaller than that of $h_{\mathrm{NaV}}$. We therefore used the classical quasi-steady-state approximation replacing $m_{\mathrm{NaV}}$ by its steady-state value $m_{\mathrm{NaV}\infty }$ (see e.g. [31]). Llinás and Muhlethaler [1] initially suggested that $I_{\mathrm{NaV}}$ in DCNn could have a persistent component. Afshari *et al*. [18] have confirmed that $I_{\mathrm{NaV}}$ in DCNn has a persistent component that they attribute to the mechanism of resurgent current initially identified in cerebellar Purkinje cells [19]. Given that the resurgent component only amounts to ${<}4\%$ of the total $I_{\mathrm{NaV}}$ in DCNn [18] and that a faithful modeling of $I_{\mathrm{NaV}}$ endowed with resurgence properties requires a set of 12 ODE [19], we chose to not introduce (see, however, the Discussion) a persistent $I_{\mathrm{NaV}}$ in our model and we rather used a classical Hodgkin–Huxley $I_{\mathrm{NaV}}$

7 $$ I_{\mathrm{NaV}} = \overline{g}_{\mathrm{NaV}}m_{\mathrm{NaV}\infty }^{3}h_{\mathrm{NaV}} ( V - E_{\mathrm{Na}} ), $$

where $m_{\mathrm{NaV}\infty }$ is given by Eq. (5) and $h_{\mathrm{NaV}}$ by Eqs. (4)–(6).

$I_{\mathrm{Kdr}}$: voltage-dependent K currents of the Kv3-type exhibit fast activation allowing ${\sim} 1~\mbox{ms}$-duration Na spikes in several neuron types [32]. Lllinás and Muhlethaler [1] report spike duration (measured at half-width) ranging from 0.44 to 0.7 ms in DCNn suggesting that spike repolarization in these neurons is achieved by Kv3 channels. Raman *et al*. [4] have dissected out a high-threshold voltage-dependent potassium current in DCNn that is be presumably involved in the repolarization of their spikes. The activation time constant of this current (12 ms at +12 mV) is too large for Kv3 channels and rather points to the Kv2 family of K channels [33]. However, Raman et al. [4] only used 1 mM TEA whereas to block voltage-dependent K channels whereas blocking Kv2 channels require several millimolar concentration of TEA to block [34]. Moreover, no molecular study has yet, to our knowledge, demonstrated expression of Kv2 channels in DCNn. On the opposite, several studies have shown that DCNn express all four Kv3 subunits (see e.g. [35] and [32]) and the electrophysiological study of Lamont [28] suggests that Kv3 channels in DCNn are functional. DCNn can fire spikes at frequencies up to ${>}100~\mbox{Hz}$ which seem unattainable with Kv2 serving to repolarize spikes. Owing to these findings, our model assumes that $I_{\mathrm{Kdr}}$ is carried by fast-activating K channels (however, see the discussion for the involvement of putative Kv2 channels),

8 $$ I_{\mathrm{Kdr}} = \overline{g}_{\mathrm{Kdr}}m_{\mathrm{Kdr}}^{4} ( V - E_{\mathrm{K}} ), $$

where $m_{\mathrm{Kdr}}$ is given by Eqs. (4)–(6). The parameters from this current were taken from a model of Purkinje cell dendrite [27].

$I_{\mathrm{TCN}}$: There is experimental evidence for a TTX-insensitive current in DCNn that can depolarize them beyond their spike threshold [4], this current being tonic (i.e. *V*-independent) and non-selective for cations [4]. We modeled this current using Ohm’s law with a constant conductance, i.e. as an effective leak current:

9 $$ I_{\mathrm{TCN}} = g_{\mathrm{TCN}} ( V - E_{\mathrm{TCN}} ). $$

*Calcium currents* $I_{\mathrm{CaH}}$ *and* $I_{\mathrm{CaT}}$: Owing to the large gradient of Ca^2+^ ions across the cytoplasmic membrane of neurons (typically $[ \mathrm{Ca} ]_{o} / [ \mathrm{Ca} ]_{i} \simeq 2 \times 10^{5}$ in a neuron at rest), voltage-dependent Ca currents are not adequately modeled by the Nernst equation. This equation accurately describes ion fluxes only close to thermodynamic equilibrium (see e.g. [26]). Far from equilibrium, Ca currents exhibit a rectification in their $I/V$ relation which is well accounted for by the constant-field equation of Goldman (see e.g. Chap. 13 in [26]). We therefore used this equation to describe Ca currents in our model. As mentioned above, two types of Ca currents have been identified in DCNn [14]: a non-inactivating high-threshold current $I_{\mathrm{CaH}}$ and an inactivating current $I_{\mathrm{CaT}}$. Both currents are given by

10 $$ I_{\mathrm{Ca}_{x}} = \frac{P_{\mathrm{Ca}_{x}} ( zF )^{2}V}{RT ( 1 - \alpha ( V ) )} \bigl( [ \mathrm{Ca} ] - \alpha ( V ) [ \mathrm{Ca} ]_{o} \bigr), $$

where $\mathrm{Ca}_{x} \in \{ \mathrm{CaT},\mathrm{CaH} \} $ and $\alpha ( V ) = e^{ - \frac{2FV}{RT}}$. A temperature $T=298~\mbox{K}$ was used in all simulations. The molecular identity of ion channels underlying $I_{\mathrm{CaH}}$ in DCNn is currently not precisely known. Gauck *et al*. [14] have shown that a large fraction of $I_{\mathrm{CaH}}$ in DCNn is non-inactivating and is therefore likely composed of L-, T- or R-type currents. They have also observed that a small fraction of $I_{\mathrm{CaH}}$ inactivates, thereby also indicating the presence of N-type calcium channels. For the sake of simplicity and since L-type calcium channels open as quickly as N-types channels in neurons [36], we derived a simple equation for $P_{\mathrm{CaH}}$ by assuming that it activates instantaneously and does not inactivate, i.e.

11 $$ P_{\mathrm{CaH}} = \overline{P}_{\mathrm{CaH}}m_{\mathrm{CaH}\infty }, $$

where $m_{\mathrm{CaH}\infty }$ is given by Eq. (5).

Setting an equation for $I_{\mathrm{CaT}}$ proved more challenging. Indeed, this current can be underlain by three isoforms of calcium channels, Cav3.1, Cav3.1 and Cav3.3 which have marked different time constants for both activation and inactivation [18]. Molineux *et al*. [25] have demonstrated the expression of Cav3.1 in both GABAergic and non-GABAergic DCNn, whereas Cav3.3 expression is restricted to non-GABAergic DCNn. Since both types of DCNn exhibit rebound properties, these findings suggest that the expression of Cav3.1 is sufficient to generate a rebound in both cell types. McKay *et al*. [20] have shown that Cav3.1 channels are largely confined to the soma of the DCNn. McRory *et al*. [30] have found that both $\tau _{\mathrm{act}}$ and $\tau _{\mathrm{inact}}$ of Cav3.1–3 channels decay monotonically with voltage toward a voltage-independent nonzero minimum. This feature is well reproduced by the thalamic $I_{\mathrm{CaT}}$ model of Destexhe *et al*. [37] which we adopted here for the DCNn. Its permeability equation reads

12 $$ P_{\mathrm{CaT}} = \overline{P}_{\mathrm{CaT}}m_{\mathrm{CaT}}^{2}h_{\mathrm{CaT}}, $$

where $m_{\mathrm{CaT}}$ and $h_{\mathrm{CaT}}$ are given by Eqs. (4)–(6). Nevertheless, we had to adapt the original model of Destexhe *et al*. [37] to provide a faithful account of $I_{\mathrm{CaT}}$ in DCNn. Firstly, we adapted the values of the time constants $\tau _{m_{\mathrm{CaT}}}$ and $\tau _{h_{\mathrm{CaT}}}$ of the original model to match the properties of the Cav3.1 channels expressed by DCNn [25]. Secondly, the model by Destexhe *et al*. uses a discontinuous piecewise function to describe $\tau _{h_{\mathrm{CaT}}}$. This prevents guarantying existence and uniqueness of the solutions of the model according to the Cauchy-Lipschitz theorem (see e.g. [38]). We therefore replaced the original formulation of $\tau_{h_{\mathrm{CaT}}}$ in Destexhe *et al*. by the continuous and differentiable formulation of Eq. (6).

$I_{\mathrm{KCa}}$: The DCNn express calcium-dependent potassium currents of the SK (small-conductance) type [25]. Those potassium-specific channels are gated by calcium according to a Hill-type equation (see e.g. [39])

13 $$ I_{\mathrm{KCa}} = g_{\mathrm{KCa}}w_{\mathrm{KCa}} ( V - E_{\mathrm{K}} ) $$

which assumes instantaneous gating of SK channels by calcium:

14 $$ w_{\mathrm{KCa}} = \frac{ [ \mathrm{Ca} ]^{5}}{C_{\mathrm{KCa}}^{5} + [ \mathrm{Ca} ]^{5}}. $$

Finally, the model includes a leakage current. The value of its conductance $g_{\mathrm{Leak}}$ was set to a value allowing the model to reproduce the passive time constant of DCNn recorded *in vitro* (as probed by small hyperpolarizing pulses)

15 $$ I_{\mathrm{Leak}} = g_{\mathrm{Leak}} ( V - E_{\mathrm{Leak}} ). $$

Table 1 lists the standard value of all parameters appearing in Eqs. (5)–(15).

### Boundedness of solutions

We follow the approach of Cronin [40] to prove that all physiologically significant solutions of the model are bounded. Let us first consider the dynamics of state variables $h_{\mathrm{NaV}}$, $m_{\mathrm{Kdr}}$, $m_{\mathrm{CaT}}$ and $h_{\mathrm{CaT}}$ which all obey ODE of the form of Eq. (4). The function $\tau _{x \in \{ h_{\mathrm{NaV}},m_{\mathrm{Kdr}},m_{\mathrm{CaT}},h_{\mathrm{CaT}} \} } ( V )$ in Eq. (4) is positive whatever the value of *V* according to Eq. (6). Moreover, for all physiologically significant initial conditions on *V* we have $0 < x < 1$ according to Eq. (5). It follows that $\frac{dx}{dt} > 0$ if $x = 0$ and $\frac{dx}{dt} < 0$ if $x = 1$. This implies that all physiological solutions remain in the

$$ \left\{ ( h_{\mathrm{NaV}},m_{\mathrm{Kdr}},m_{\mathrm{CaT}}, m_{\mathrm{CaT}} )\left| \textstyle\begin{array}{c} h_{\mathrm{NaV}} \\ m_{Kdr} \\ m_{CaT} \\ m_{CaT} \end{array}\displaystyle \right. \in [ 0,1 ] \right\} $$

region of the model phase space.

Let us now turn to Eq. (1) in the particular case where $P_{\mathrm{CaH}} = P_{\mathrm{CaT}} = 0$ (namely the model deprived of its Ca currents) and let $E_{\max } = \max \{ E_{\mathrm{Na}},E_{\mathrm{K}},E_{\mathrm{Leak}} \} $ and $E_{\min } = \min \{ E_{\mathrm{Na}},E_{\mathrm{K}},E_{\mathrm{Leak}} \} $. Recall then that currents $I_{\mathrm{Na}}$, $E_{\mathrm{K}}$ and $E_{\mathrm{Leak}}$ all have the form $I_{i} = f_{i} ( V ) ( V - E_{i} )$, $i \in \{ \mathrm{Na},\mathrm{K},\mathrm{Leak} \} $ with $0 < f_{i} ( V ) < 1$. Then Eq. (1) with $I_{S} = 0$ implies that $\frac{dV}{dt} > 0$ if $V < E_{\min } $ and that $\frac{dV}{dt} < 0$ if $V > E_{\max } $. It follows that all physiological solutions remain in the

$$ \left\{ ( V,h_{\mathrm{NaV}},m_{\mathrm{Kdr}}, m_{\mathrm{CaT}},h_{\mathrm{CaT}} )\left|V \in [ E_{\min },E_{\max } ],\textstyle\begin{array}{c} h_{\mathrm{NaV}} \\ m_{\mathrm{Kdr}} \\ m_{\mathrm{CaT}} \\ h_{\mathrm{CaT}} \end{array}\displaystyle \right. \in [ 0,1 ] \right\} $$

subset of the phase space.

Proof of the boundedness of solutions is finalized by extending the above result to the full model (i.e. including Ca currents) as follows. Let now $E_{\max } = \max \{ E_{\mathrm{Na}},E_{\mathrm{K}},E_{\mathrm{Leak}},E_{\mathrm{Ca}} \} $ and $E_{\min } = \min \{ E_{\mathrm{Na}},E_{\mathrm{K}},E_{\mathrm{Leak}},E_{\mathrm{Ca}} \} $. The factor $\alpha ( V )$ in Eq. (10) of the Ca currents is easily shown to be positive whatever the value of *V*. The sign of the $[ \mathrm{Ca} ] - \alpha ( V ) [ \mathrm{Ca} ]_{o}$ factor depends on both *V* and $[ \mathrm{Ca} ]$. Nevertheless, it is easily shown that $I_{\mathrm{Ca}_{x}} < 0$ for $\{ ( V, [ \mathrm{Ca} ] )|V < E_{\mathrm{Ca}}^{\max }, [ \mathrm{Ca} ]_{b} < [ \mathrm{Ca} ] < [ \mathrm{Ca} ]_{0} \} $ and positive for $\{ ( V, [ \mathrm{Ca} ] )|V > E_{\mathrm{Ca}}^{\max }, [ \mathrm{Ca} ]_{b} < [ \mathrm{Ca} ] < [ \mathrm{Ca} ]_{0} \} $ where $E_{\mathrm{Ca}}^{\max } = \frac{RT}{2F}\ln \frac{ [ \mathrm{Ca} ]_{0}}{ [ \mathrm{Ca} ]_{b}}$. According to Eq. (2), it follows from this last result that $\frac{d [ \mathrm{Ca} ]}{dt} > 0$ if $[ \mathrm{Ca} ] < [ \mathrm{Ca} ]_{b}$ and $\frac{d [ \mathrm{Ca} ]}{dt} < 0$ if $[ \mathrm{Ca} ] > [ \mathrm{Ca} ]_{0}$ and therefore that physiologically solutions of the model stay in the set

$$\begin{aligned}& \left\{ ( V,\mathrm{Ca},h_{\mathrm{NaV}},m_{\mathrm{Kdr}}, m_{\mathrm{CaT}},h_{\mathrm{CaT}} )\left|V \in [ E_{\min },E_{\max } ],\vphantom{\textstyle\begin{array}{c} h_{\mathrm{NaV}} \\ m_{\mathrm{Kdr}} \\ m_{\mathrm{CaT}} \\ h_{\mathrm{CaT}} \end{array}\displaystyle }\right.\right. \\& \quad \left.[ \mathrm{Ca} ] \in \bigl[ [ \mathrm{Ca} ]_{b}, [ \mathrm{Ca} ]_{0} \bigr],\textstyle\begin{array}{c} h_{\mathrm{NaV}} \\ m_{\mathrm{Kdr}} \\ m_{\mathrm{CaT}} \\ h_{\mathrm{CaT}} \end{array}\displaystyle \in [ 0,1 ] \right\} . \end{aligned}$$

## Results

### Theoretical evidence for two stable states of electric activity in DCNn

**Figure 1:**
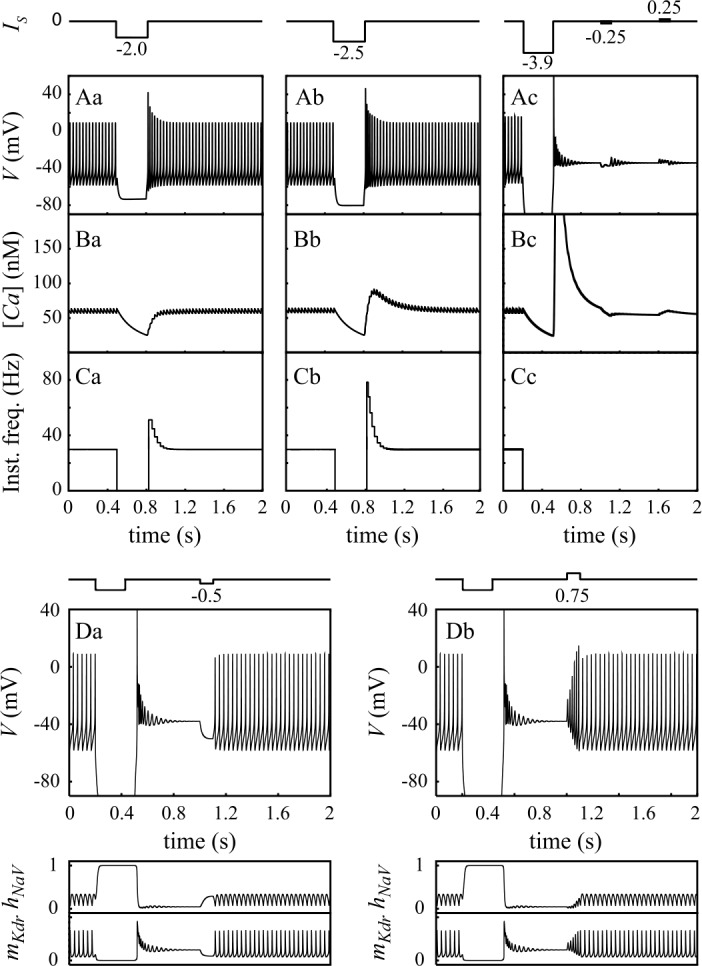
A–C. Basic I/O relationship of the model probed with injected pulses of hyperpolarizing current (300 ms duration). Amplitudes from left to right: $-2\times 10^{3}$, $-2.5 \times 10^{3}$ and $-3.9 \times 10^{3}~\mbox{nAcm}^{-2}$. A: membrane potential. B: cytoplasmic free Ca^2+^ concentration [Ca]. C: instantaneous spiking frequency. D. Transition of the model from silent mode to firing mode triggered by hyperpolarizing (D_1_, $-0.5 \times 10^{3}~\mbox{nAcm}^{-2}$) and depolarizing current pulses (D_2_, $0.75 \times 10^{3}~\mbox{Acm}^{-2}$). Bottom: corresponding time course of the $h_{\text{NaV}}$ ($I_{\text{Na}}$ inactivation) and $m_{\text{Kdr}}$ ($I_{\text{Kdr}}$ activation) variables

Consistent experimental data show that DCNn are pacemaker neurons *in vitro* [2–7]. In the absence of a depolarizing bias current ($I_{\mathrm{DC}} = 0$), our neuron model successfully reproduces this property, with the spontaneous emission of self-sustained fast (${\sim} 1~\mbox{ms}$ duration) spikes (Fig. 1A_1_). We refer to this mode of activity as the firing (F) mode. The spontaneous firing frequency in the model is 28.9 Hz (versus 26 Hz in [2] and ${\sim}20~\mbox{Hz}$ in [4]). The amplitude of spontaneous spikes is 67 mV (in the range of extreme values reported experimentally: $57\pm 5~\mbox{mV}$ in [1] and 82 mV in [4]). As shown in Fig. 1A_1_, a 300 ms hyperpolarizing pulse of $-2~\mu \mbox{Acm}^{-2}$ amplitude interrupts spike firing in the model. Firing resumes at the end of the pulse with an initial phase of increased firing frequency of ${\sim}200~\mbox{ms}$ duration (Fig. 1A_1_ and C_1_). The concentration of free cytosolic calcium $[ \mathrm{Ca} ]$ first decreases during the pulse and quickly recovers its baseline level after the end of the pulse (Fig. 1B_1_). Increasing the pulse amplitude shows non-monotonous effects. A moderate increase of the amplitude of the pulse (to e.g. $-2.5~\mu \mbox{Acm}^{-2}$, Fig. 1A_2_–C_2_) yields the same behavior, but with a larger transient firing frequency increase at pulse break. The peak frequency during this rebound is close to twice the basal frequency (Fig. 1C_2_), in close agreement with the experimental data of Raman *et al*. [4]. We also note the appearance of a rebound $[ \mathrm{Ca} ]$ increase at the pulse break, which is consistent with the observations of Raman et al. [4] (Fig. 1B_2_). Large pulse amplitudes, however, produce a dramatic change of response (Fig. 1A_3_–C_3_). For instance, a $-3.9~\mu \mbox{Acm}^{-2}$ pulse triggers a switch of the model to a silent depolarized (SD) state in which the membrane potential settles at −38 mV (Fig. 1A_3_). This model feature reproduces the observation of Jahnsen ([2]; see Fig. 5C) that a hyperpolarizing current pulse can switch spontaneously firing DCNn to a silent mode of activity. In the model, the SD state is stable as evidenced by the fact that, once settled in this state, the model remains in this state despite small depolarizing or hyperpolarizing pulses of current (Fig. 1A_3_). However, this stability is only local since larger-amplitude hyperpolarizing current pulses (Fig. 1D_1_) readily switch the model back to its firing mode. This new behavior is also consistent with the following experimental result by Jahnsen ([2]; see Fig. 5E): when DCNn are switched to the SD state, a subsequent hyperpolarizing pulse can switch them back to the F mode. However, our model extends this finding by showing that depolarizing current should also be able to trigger this transition (Fig. 1D_2_). Therefore, our model reproduces the experimental observations that hyperpolarizing pulses can switch the DCNn from their spontaneous low frequency firing (F) state to a stable depolarized (SD) state. The SD state is only locally stable and injection of large hyperpolarizing or depolarizing current pulses switches the DCNn model back to its F state. In the following we provide a mathematical analysis of the model in order to dissect the contribution of the different membrane ion currents to these dynamical features.

### Bifurcation analysis of the model and the silent depolarized state (SD)

Bifurcation analysis provides an explanatory scheme for the emergence of the overall properties of a dynamical system (see e.g. [41] for an introduction). In bifurcation analysis of neuron models, the steady injected current ($I_{\mathrm{DC}}$ in our model) is the most important bifurcation parameter as it is used in experiments as a model of synaptic inputs to investigate the electric properties of the neuron. The power of this approach was first demonstrated by Rinzel and Ermentrout [41] who showed that the type I and II responses identified by Hodgkin [42] in crab neurons are due to different bifurcations (see Izhikevich [43] for a comprehensive exposure of the geometry of excitability of nerve cells). We therefore followed this approach and we built bifurcation diagrams of the model with $I_{\mathrm{DC}}$ as the bifurcation parameter.

**Figure 2:**
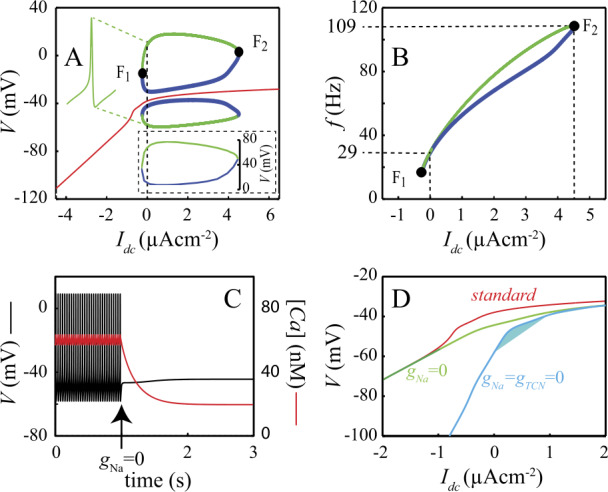
Bifurcation analysis of the standard model with $I_{\text{DC}}$ as the bifurcation parameter. A. Red: branch of stable fixed points (FP). Green: envelop (min and max) of a branch of stable limit cycles (sLC). Blue: envelop of a branch of unstable limit cycles (uLC). sLC and uLC appear as a fold of limit cycle (bifurcation $F_{1}$) and disappear in another fold of limit cycle (bifurcation $F_{2}$). Inset: amplitude (mV) of spikes corresponding to the branches of sLC and uLC. B. Firing frequency of stable and unstable spikes as a function of $I_{\text{DC}}$. C. *V* (black) and [Ca] (red) trajectories following blocking of $I_{\text{NaV}}$. D. The FP branch in the standard model (red) is altered by blocking $I_{\text{NaV}}$ (green) and blocking $I_{\text{NaV}}$ and $I_{\text{TCN}}$ (blue)

In the presence of hyperpolarizing bias currents, the model has a unique stable attractor, the SD state (Fig. 2A). This state is actually stable in the global sense (i.e. with respect to any perturbation whatever its amplitude) since it is the only attractor in the phase space. Firing arises upon increasing the bias current $I_{\mathrm{DC}}$ to $I_{F_{1}}=-255.3~\mbox{nAcm}^{-2}$, a value at which a unique neutrally stable periodic orbit appears (Fig. 2A). Two different branches of Limit Cycles (LC) emerge from this orbit: one of them (green) corresponds to stable LC (sLC) whereas the other one corresponds to unstable LC (uLC). This type of bifurcation is usually referred to as fold bifurcation of LC. The neutrally stable LC has a frequency of 17.8 Hz at the $F_{1}$ bifurcation point (Fig. 2B). These results suggest that the DCNn are type II neurons characterized by both a nonzero frequency and a nonzero spike amplitude at the bifurcation point [38]. Upon increasing $I_{\mathrm{DC}}$, the amplitude of the sLC steeply increases while that of the uLC first decreases, exhibiting a minimum at $I_{\mathrm{DC}} \simeq 1000~\mbox{nAcm}^{-2}$ (inset Fig. 2A), and increases afterwards. Likewise, the amplitude of the sLC shows a maximum at $I_{\mathrm{DC}} \simeq 1000~\mbox{nAcm}^{-2}$ then decreases beyond this value. The parallel decrease of the amplitude of sLC and increase of the amplitude of uLC ultimately results in the merging of the two LC branches into another neutrally stable LC through a second fold bifurcation at $I_{F_{2}}=4256~\mbox{nAcm}^{-2}$ (Fig. 2A). The theoretical maximum firing frequency of DCNn occurs at $I_{F_{2}}$ and is 108 Hz, in the range of experimental maximum frequencies (80–120 Hz [7]). The branches of sLC and uLC thereby form a closed loop of limit cycles solutions of the model which is usually referred to as an isola of limit cycles (see e.g. Avitabile *et al*. [44] and Labouriau [45, 46] in the case of the HH model). This isola coexists with a branch of stable FP. Figure 2A thereby suggests that DCNn could switch between silent and firing modes of activity within the range of current $[ I_{F_{1}},I_{F_{2}} ]$ in response to proper inputs. Spontaneous firing in the model disappears upon zeroing $g_{\mathrm{NaV}}$ (Fig. 2C). This result reproduces the finding that $I_{\mathrm{Na}}$ underlies the rising phase of the spike in the DCNn [4]. After $g_{\mathrm{NaV}}$ is zeroed, the membrane potential settles to a stable state with membrane potential $V=-44.4~\mbox{mV}$, i.e. above the spike undershoot ($V_{U}=-58.1~\mbox{mV}$). This result is at odds with properties of all neuron types studied so far (to the best of our knowledge) of which the membrane potential converges to values below $V_{U}$ after blocking of the Na currents with TTX. Nevertheless, this model feature closely reproduces the experimental observation that, after blocking $I_{\mathrm{NaV}}$ with TTX, the DCNn settle to a depolarized stable voltage of $-42\pm 2~\mbox{mV}$ [4]. This property is well explained by the finding that $I_{\mathrm{TCN}}$ provides a depolarizing current to the membrane, as illustrated in Fig. 2D which shows that the branch of FP is shifted downright when $I_{\mathrm{TCN}}$ is blocked.

**Figure 3:**
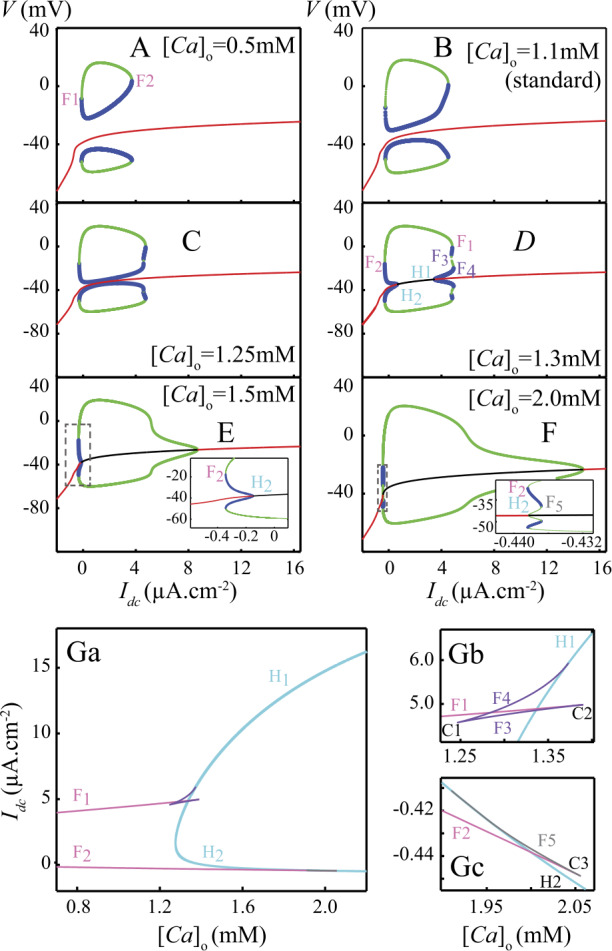
Extracellular calcium controls the emergence of the isola of limit cycles. A–F: bifurcation diagram of the model with $I_{\text{DC}}$ as the bifurcation parameter for different external Ca^2+^ concentrations ($[ \text{Ca} ]_{0}$). The color-code is the same as in Fig. 2, in particular for the envelope of the sLC (green), the uLC (blue) and the stable branch of fixed points FP (red). Around 1.3 mM extracellular Ca (panel D), the uLC collides with FP thus creating two Hopf bifurcation points ($H_{1}$ and $H_{2}$). Bifurcation $F_{2}$ is also altered by the appearance of two additional folds of limit cycles ($F_{3}$ and $F_{4}$). $F_{1}$ is also eventually modified by the appearance of another fold of limit cycles ($F_{5}$). Panels in (G) show the corresponding two-parameter bifurcation diagrams: G_1_ is the overall diagram while G_1_ and G_2_ show magnifications of G_1_ around $[ \text{Ca} ]_{0} = 1.3$ and 2.0 mM, respectively. The curves show how the bifurcation points described in A–F change when $[ \text{Ca} ]_{0}$ or $I_{\text{DC}}$ are varied. The branch of folds $F_{1}$ and $F_{2}$ are shown in pink and the two branches corresponding to the Hopf bifurcations $H_{1}$ and $H_{2}$ are shown in cyan. The additional folds associated to $F_{1}$ ($F_{3}$ and $F_{4}$) are shown in purple and the additional fold that is associated to $F_{2}$ ($F_{5}$), is in black. Those Fold branches appear and disappear by collisions with each other at codimension-2 bifurcation points called cusps of limit cycles, show as points *C*. Thus, $F_{3}$ and $F_{4}$ collides at cusp $C_{1}$, $F_{3}$ and $F_{1}$ at $C_{2}$, and $F_{2}$ and $F_{5}$ at $C_{5}$

The report of a bistability between an isola of LC and a stable FP branch in a neuron membrane model is not unprecedented. Guttman *et al*. [12] have described similar dynamics in a model of the squid axon, which exhibits a stable point attractor coexisting with a stable firing mode of spikes when bathed in low $[ \mathrm{Ca} ]_{0}$ solutions. This prompted us to investigate the hypothesis that $[ \mathrm{Ca} ]_{0}$ may control bistability in DCNn by studying how $[ \mathrm{Ca} ]_{0}$ affects the bifurcation diagram of the model (Fig. 3). With 0.5 mM external Ca^2+^ (Fig. 3A), the isola is characterized by the coexistence of a stable fixed point (red), an unstable limit cycle (min-max values in blue) and a stable limit cycle (min-max values in green) within the range $I_{\mathrm{DC}} = [0,4]~\mu \mbox{A/cm}^{2}$. Two folds of limit cycles ($F_{1}$ and $F_{2}$, pink in Fig. 3G) locate the coalescence of the two limit cycles. With increasing external Ca^2+^, the unstable limit cycle gets closer to the fixed point (Fig. 3B, C), colliding it for $[ \mathrm{Ca} ]_{0} \simeq 1.26~\mbox{mM}$ (Fig. 3C). The collision with the fixed point gives birth to (Fig. 3G_1_) two subcritical Hopf bifurcations ($H_{1}$ and $H_{2}$, blue in G) between which the fixed point becomes unstable (black in D). In addition, a pair of limit cycles (one stable, one unstable) appears by a cusp of LC ($C_{1}$ in Fig. 3G_2_), thus giving rise to two new folds of LC ($F_{3}$ and $F_{4}$, purple in Fig. 3G_2_). $F_{4}$ disappears by coalescence with $H_{1}$ and $F_{3}$ with $F_{1}$ in the cusp of limit cycles $C_{2}$ (Fig. 3G_2_), leaving $H_{1}$ as the only bifurcation point in the zone for $[ \mathrm{Ca} ]_{0}>1.4~\mbox{mM}$ (Fig. 3E). At even larger $[ \mathrm{Ca} ]_{0}$ (around 2 mM), a new stable LC appears close to $F_{2}$ and $H_{2}$ as a result of $H_{2}$ becoming supercritical, giving rise to $F_{5}$ (Fig. 3F, gray in Fig. 3G_3_). $F_{2}$ and $F_{5}$ in turn disappear by coalescence at the cusp of limit cycles $C_{3}$ (Fig. 3G_3_), leaving $H_{2}$ as the only bifurcation point in the zone for $[ \mathrm{Ca} ]_{0}>2.1~\mbox{mM}$ (Fig. 3G_1_). These results therefore suggest the existence of a bistability (stable fixed point SD + stable limit cycle sLC) at physiological $[ \mathrm{Ca} ]_{0}$ in the electro-responsiveness of DCNn.

### Reversible $\text{F} \leftrightarrow \text{DB}$ transitions induced by blocking $I_{\text{CaH}}$: role of the separatrix between the F and SD states

**Figure 4:**
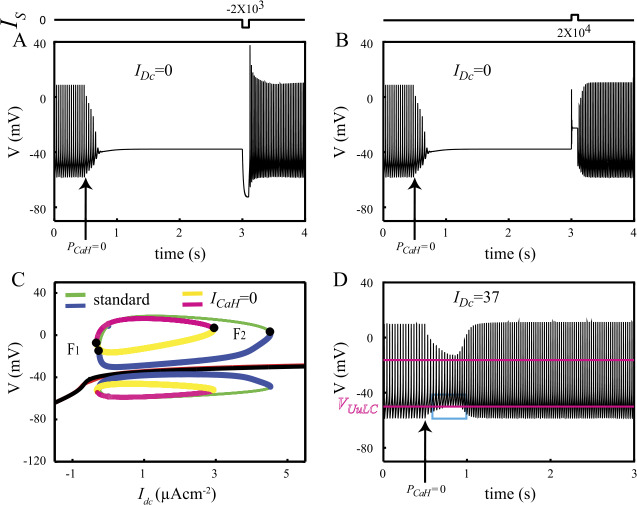
Reversible $\text{F} \leftrightarrow \text{SD}$ state transitions induced by blocking $I_{\text{CaH}}$. A. Blocking $I_{\text{CaH}}$ switches the model from its F to its SD state. Spike firing resumes in response to a hyperpolarizing pulse of injected current ($-2\times 10^{3}~\mbox{nAcm}^{-2}$, 100 ms). B. The model can also be switched back to its firing mode by a depolarizing pulse ($2 \times 10^{4}~\mbox{nAcm}^{-2}$, 100 ms). C. Bifurcation diagrams in the standard model and after blocking $I_{\text{CaH}}$ show that coexistence of the branch of FP and the isola of LC remains after blocking $I_{\text{CaH}}$. Blocking $I_{\text{CaH}}$ induces the $\text{F}\to \text{SD}$ transition by reducing the attraction basin of the sLC (see text). D. In the presence of a depolarizing bias current, blocking $I_{\text{CaH}}$ fails to trigger the $\text{F} \to \text{SD}$ transition, despite the membrane potential transiently goes above the undershoot of the uLC, $V_{U_{\text{uLC}}}$ (blue box)

Raman *et al*. [4] report that blocking Ca currents of DCNn switches their spontaneously firing activity to a silent depolarized mode at $-37~\mbox{mV}$. Our model reproduces this experimental result as it switches onto a silent depolarized state (SD) after blocking both of its Ca currents, $I_{\mathrm{CaH}}$ and $I_{\mathrm{CaT}}$ (not illustrated). The membrane voltage adopted by our model ($-38~\mbox{mV}$) is very close to the experimental value reported by Raman *et al*. [4]. The experimental protocol of Raman *et al*. prevented them to determine whether the $\mathrm{F} \to \mathrm{SD}$ switch results from blocking both Ca currents or from blocking only one of them because they blocked both currents with 2 mM cobalt (see [47]). We could readily address this question with the model. Blocking $I_{\mathrm{CaT}}$ (i.e. setting $P_{\mathrm{CaT}}=0$) does not induce a $\mathrm{F} \to \mathrm{SD}$ transition (result not shown). This finding is not surprising since $I_{\mathrm{CaT}}$ is almost completely inactivated at the spikes undershoot voltage ($h_{\mathrm{CaT}} \simeq 5.5 \times 10^{ - 4}$), which is the more negative *V* value spontaneously reached by the model in its F mode. On the opposite, blocking $I_{\mathrm{CaH}}$ induces a $\mathrm{F} \to \mathrm{SD}$ transition (Fig. 4A). Following this switch, brief pulses of hyper- (Fig. 4A) or depolarizing (Fig. 4B) injected current reset firing in the model. Figure 4C explains this finding by showing that both the branch of FP and the isola of LC of the standard model are preserved after blocking $I_{\mathrm{CaH}}$, thereby allowing theoretical $\mathrm{F} \leftrightarrow \mathrm{SD}$ state transitions. We now give evidence that the mechanism of the $\mathrm{F} \to \mathrm{SD}$ transition induced by blocking $I_{\mathrm{CaH}}$ finds its origin in the modifications of the shape of the isola of LC illustrated in Fig. 4C. Recall from Sect. 1 that the SD state is only locally stable. It therefore has a basin of attraction that is the set of initial conditions from which the model converges onto the SD state. However, in the bistable regime the coexisting sLC also has its own basin of attraction. Cooley et al. [8] early conjectured that for membrane models with only two variables, this situation implicates the existence of an uLC separating the basins of attraction of the two stable states. In other words, the separatrix between the basins of attraction of the SD and the F states in two-dimensional models is the orbit of the uLC in the phase plane. In $n > 2$ dimension models the separatrix must be a manifold of higher dimension than the one-dimensional uLC since the separatrix partitions the phase space into two attraction basins. Guttman *et al*. [12] have hypothesized that the separatrix contains the uLC and it is reasonable to suppose that it is a $n - 1$ dimension manifold. To our knowledge, neither of these two conjectures have been proved until now. Assuming that they are true (see the appendix), we propose that blocking $I_{\mathrm{CaH}}$ in our six-dimensional model triggers the $\mathrm{F} \to \mathrm{DB}$ transition by reducing the maximum amplitude of the uLC (compare blue and yellow undershoot voltages of the uLC in Fig. 4C). This shrinks the attraction basin of the sLC at such point that a trajectory located in the basin of attraction of the sLC before blocking $I_{\mathrm{CaH}}$ finds itself on the other side of the separatrix (inside the basin of attraction of the FP) when $I_{\mathrm{CaH}}$ is blocked and eventually converge to the SD state. When a small tonic current ($I_{\mathrm{DC}}=37~\mbox{nAcm}^{-2}$) is injected, blocking $I_{\mathrm{CaH}}$ is no more able to trigger the $\mathrm{F} \to \mathrm{SD}$ transition although the membrane voltage crosses several times the undershoot voltage of the uLC (Fig. 4D). This confirms that the separatrix between the F and SD attraction basins does not reduce to the uLC and is rather a manifold of larger dimension. We provide further evidence for this conjecture and that of Guttman *et al*. [12] in the Appendix. Taken together these results suggest that the coexistence of a branch of stable silent states with an isola of LC in the DCNn does not rest on the expression of Ca currents by the DCNn. $I_{\mathrm{KCa}}$ cannot play a central role in these dynamical features since it is almost completely inactivated at the low $[ \mathrm{Ca} ]$ level reached by the model when the Ca currents are blocked ($m_{\mathrm{KCa}} \simeq 10^{ - 3}$ at $[ \mathrm{Ca} ] \simeq 50~\mbox{nM}$). The origin of the peculiar bifurcation diagram of the standard model illustrated in Fig. 2A has therefore to be searched in the interactions between the remaining active currents in the model, namely $I_{\mathrm{Na}}$, $I_{\mathrm{Kdr}}$ and $I_{\mathrm{TCN}}$. This question is addressed after the following section, in which we expose the role played by $I_{\mathrm{CaT}}$ in the $\mathrm{F} \to \mathrm{SD}$ transitions triggered by hyperpolarizing current pulses in the standard model.

### A mechanism for $\text{F} \to \text{SD}$ transitions triggered by current injections: the role of $I_{\text{CaT}}$

**Figure 5:**
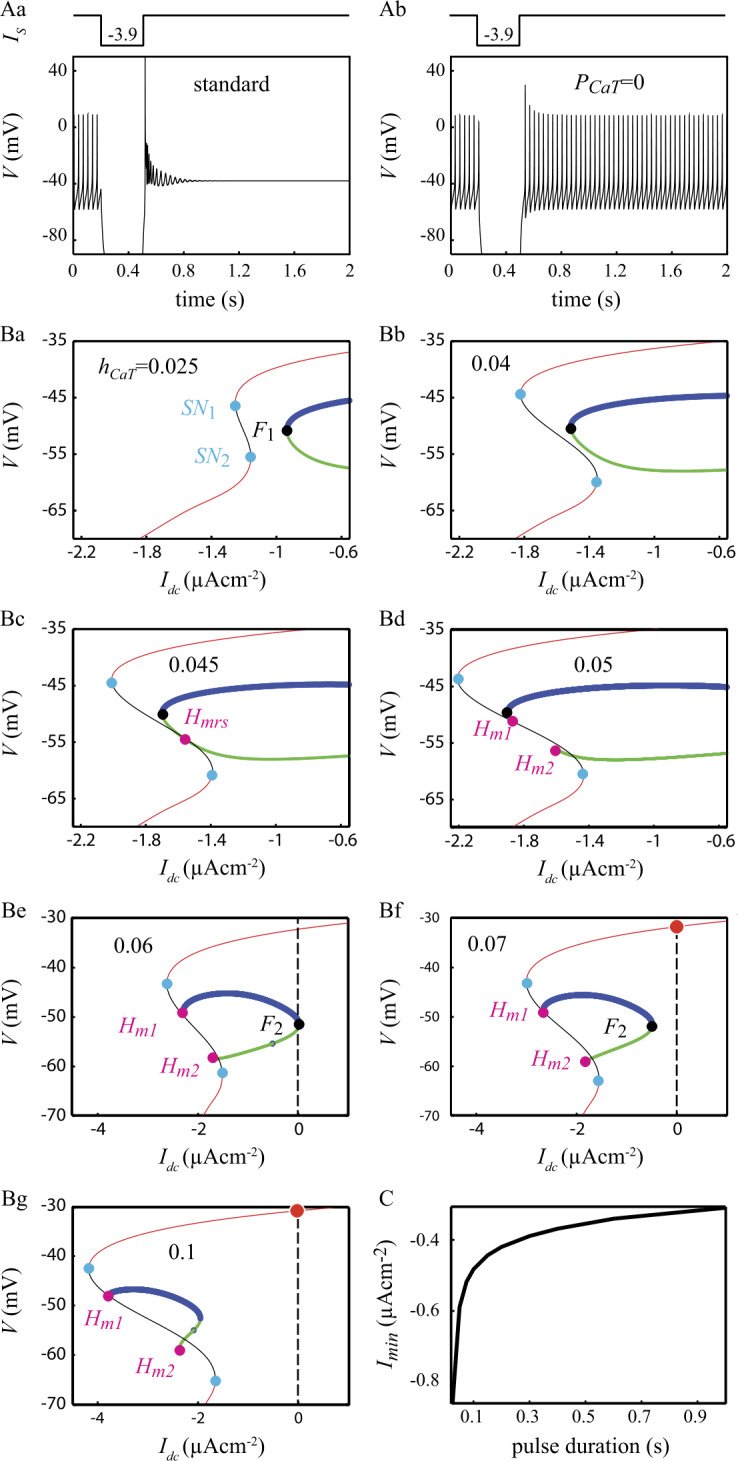
Contribution of $I_{\mathrm{CaT}}$ to $\text{F} \to \text{SD}$ transitions triggered by hyperpolarizing current pulses. A. Response of the standard model to a 300 ms hyperpolarizing pulse ($-3.9~\mu \mbox{Acm}^{-2}$) before (A_1_) and after (A_2_) blocking $I_{\mathrm{CaT}}$. B. Evolution of the bifurcation diagram of a variant model where the inactivation variable $h_{\mathrm{CaT}}$ is considered an adjustable parameter. C. Minimum amplitude of a hyperpolarizing current pulse capable to trigger the $\text{F} \to \text{SD}$ transition as a function of the pulse duration

Figure 5A_1_ (reproducing Fig. 1A_1_ above) recalls that large hyperpolarizing current pulses can trigger a $\mathrm{F} \to \mathrm{SD}$ transition in the model. However, Fig. 5A_2_ shows that the same current pulse fails to induce this transition if $I_{\mathrm{CaT}}$ is blocked. This result evidences the crucial role played by $I_{\mathrm{CaT}}$ in deciding whether a hyperpolarizing input can trigger a $\mathrm{F} \to \mathrm{SD}$ transition. Understanding the impact of $I_{\mathrm{CaT}}$ was complicated by the fact that both the steady-state value $h^{\infty}_{\mathrm{CaT}}$ and the time constant $\tau _{h_{{\mathrm{CaT}}}}$ of the inactivation variable $h_{\mathrm{CaT}}$ of $I_{\mathrm{CaT}}$ are voltage-dependent so that both the magnitude and the duration of the current pulses determine the amount of recruited $I_{\mathrm{CaT}}$ (see Eq. (12)). We first investigated whether long duration current pulses (i.e. of duration $\gg\tau _{h_{{\mathrm{CaT}}}}$) are able to trigger a $\mathrm{F} \to \mathrm{SD}$ transition in a variant model in which $h_{\mathrm{CaT}}$ is no longer a function of time and membrane voltage but a constant value freely adjustable in the $[0,1]$ continuous interval. With $h_{\mathrm{CaT}} = 0$, the bifurcation diagram of this variant model is nearly identical to that of the standard model (not illustrated). Increasing $h_{\mathrm{CaT}}$ to 0.025 induces a loss of stability of the FP branch with the appearance of two saddle-node bifurcations, $SN_{1}$ and $SN_{2}$ (Fig. 5Ba). The FP branch is stable below $I_{SN_{1}}$ and above $I_{SN_{2}}$. The model is hence bistable for $I_{\mathrm{DC}} \in [ I_{SN_{1}},I_{SN_{2}} ]$, the unstable intermediate branch of FP points (black in Fig. 5B_1_) delimiting the attraction basins of the two stable FP states. The gap between the two saddle-node bifurcations widens when $h_{\mathrm{CaT}}$ is increased (Fig. 5B_2_). In addition, the $F_{1}$ limit of the isola of LC is shifted leftward so that $I_{F_{1}} \in [ I_{SN_{1}},I_{SN_{2}} ]$: the model becomes tristable in this range of injected currents (two stable silent FP states and a stable LC). Both modifications of the bifurcation diagram go on as $h_{\mathrm{CaT}}$ is further increased up to 0.045 where the branch of uLC collides the unstable branch of FP points resulting in a homoclinic bifurcation of uLCs (Fig. 5B_3_). Above this $h_{\mathrm{CaT}}$ value, another bifurcation occurs with the homoclinic bifurcation splitting into two homoclinic bifurcations, $H_{m1}$ and $H_{m2}$ (see Fig. 5B_4_ for $h_{\mathrm{CaT}}=0.05$). Both bifurcations correspond to the collision of homoclinic orbits (corresponding to uLCs with a frequency equal to zero) with the unstable branch of FP. But as $h_{\mathrm{CaT}}$ keeps increasing, the $F_{1}$ limit of the isola keeps shifting to the left and the $H_{m1}$ bifurcation turns into a homoclinic bifurcation of sLC at saddle node (for $h_{\mathrm{CaT}}=0.07$, Fig. 5B_5_). In parallel with these effects on the left-hand side of the isola, increasing $h_{\mathrm{CaT}}$ also shifts the $F_{2}$ bifurcation to the left (see the sequence of Fig. 5B$_{1\text{--}6}$). Above $h_{\mathrm{CaT}}=0.06$, $I_{F_{2}}<0$ so that the only attractor remaining in the phase space for $I_{\mathrm{DC}}=0$ is the SD state (solid red circle in Fig. 5B$_{6\text{--}7}$) and the model is therefore forced to converge onto this SD state. We therefore conclude that hyperpolarizing current pulses trigger the $\mathrm{F} \to \mathrm{DB}$ transition because they de-inactivate $I_{\mathrm{CaT}}$ which leads to the disappearance of the isola of LC. In support of this conclusion, Fig. 5C shows that the minimum amplitude of a hyperpolarizing pulse to trigger the $\mathrm{F} \to \mathrm{DB}$ transition decreases with the pulse duration, in agreement with the fact that having longer duration pulses allows for recruiting larger fractions of $I_{\mathrm{CaT}}$ owing to the non-instantaneous activation of this current.

### Mechanism of $\text{SD} \to \text{F}$ transitions triggered by current injections: crossing the separatrix

From a biological standpoint, the hypothesis of the existence of a silent depolarized state only holds if the DCNn in the SD state are one way or another capable to switch back to their F mode. Otherwise, the entire population of DCNn would eventually become trapped to the SD state. As this prediction contradicts observations of active DCNn in fully developed organisms, our model had (i) to provide evidence for physiological stimuli capable to trigger $\mathrm{SD} \to \mathrm{F}$ transitions and (ii) explain the mechanism of these transitions in order to sustain the hypothesis of the SD state in DCNn. Our standard model does produce such $\mathrm{SD} \to \mathrm{F}$ transitions when hyperpolarizing (Fig. 1D_1_) or depolarizing pulses (Fig. 1D_2_) are injected. We next studied the mechanism of this transition.

**Figure 6:**
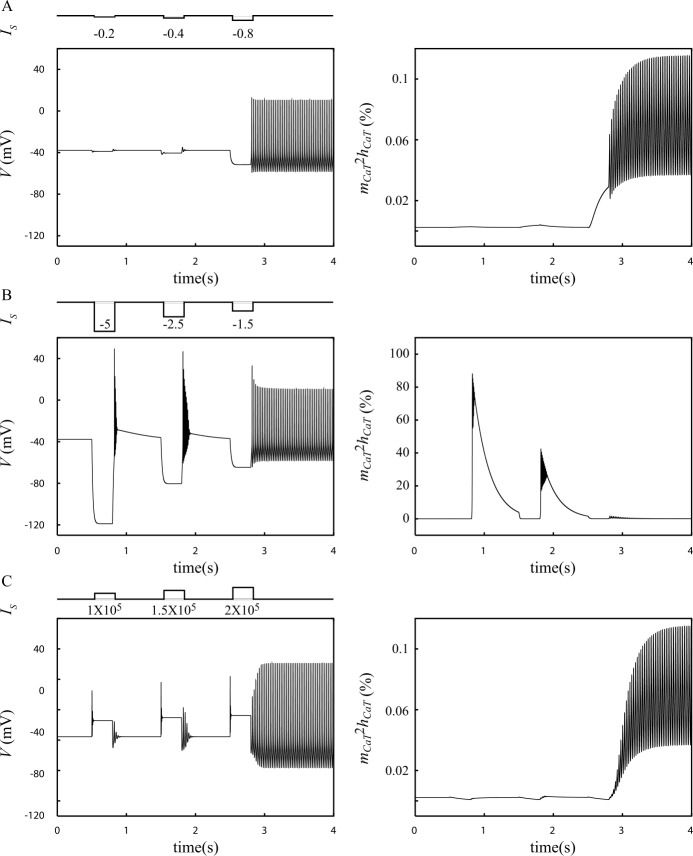
Mechanism of the $\text{SD} \to \text{F}$ transition with pulses of injected currents. The model is initially in the SD state. Pulses of injected current of various amplitude are then used to probe the switch of the model from the SD to the F mode. A. Small hyperpolarizing pulses. B. Large hyperpolarizing pulses. C. Depolarizing pulses. Left: membrane voltage (*V*) responses to pulses. Right: percentage activation of the $I_{\text{CaT}}$ current

Figure 6A shows that a hyperpolarizing pulse must have a minimum amplitude in order to trigger a $\mathrm{SD} \to \mathrm{F}$ transition. However, this minimum amplitude is clearly not a simple threshold value since Fig. 6B shows that if its amplitude is too large, a hyperpolarizing pulse fails to trigger the switch because it de-inactivates $I_{\mathrm{CaT}}$ (Fig. 6B, the first two pulses are large enough to activate 40 to 80% of this current). On the other hand, the mechanism of the $\mathrm{SD} \to \mathrm{F}$ transition triggered by pulses of moderate amplitude like the third pulse in Fig. 6A cannot involve $I_{\mathrm{CaT}}$. Indeed, this current remains inactivated during and after the pulse (<0.15 % activation, Fig. 6A) so one does not expect significant changes in the bifurcation diagram due to $I_{\mathrm{CaT}}$ deinactivation. In this case, the transition can only be explained by the fact that hyperpolarizing pulses of moderate amplitude bring the trajectory of the model across the separatrix between the F and SD attraction basins. After entering the F state attraction basin, the model eventually converges to this state. Therefore, to trigger a $\mathrm{SD} \to \mathrm{F}$ transition in the model, the amplitude of a hyperpolarizing pulse must be within an effective range: it must be large enough to drive the system on the other side of the $\mathrm{F}\text{--}\mathrm{SD}$ separatrix but weak enough so as not to de-inactivate $I_{\mathrm{CaT}}$.

A fundamental consequence of the above analysis is the prediction that any perturbation driving only once the trajectory across the separatrix should be able to trigger a $\mathrm{SD} \to \mathrm{F}$ transition. The result illustrated in Fig. 6C confirms this prediction as it shows that not only hyperpolarizing currents but also depolarizing current pulses can trigger a $\mathrm{SD} \to \mathrm{F}$ transition. Altogether, these results support the hypothesis that crossing the separatrix between the basins of attraction of the F and SD state is the basic mechanism by which phasic inputs, either depolarizing or hyperpolarizing, trigger a $\mathrm{SD} \to \mathrm{F}$ state transition. As shown above, altering the calcium currents induces qualitative changes of the bifurcation structure (i.e. changes of the size of the basins of attraction or translations of the bifurcation points), but preserves the overall structure, i.e. the coexistence of a branch of stable silent states with an isola of limit cycles. Therefore, the origin of the peculiar bifurcation diagram of the standard model illustrated in Fig. 2A does not rest on the expression of Ca currents in the DCNn but has to be searched in the interactions between the remaining active currents in the model, namely $I_{\mathrm{NaV}}$, $I_{\mathrm{Kdr}}$ and $I_{\mathrm{TCN}}$.

### The $\{ V,m_{\text{Kdr}},h_{\text{NaV}} \} $ sub-system and the role of $I_{\text{TCN}}$ in stabilizing the SD state

**Figure 7:**
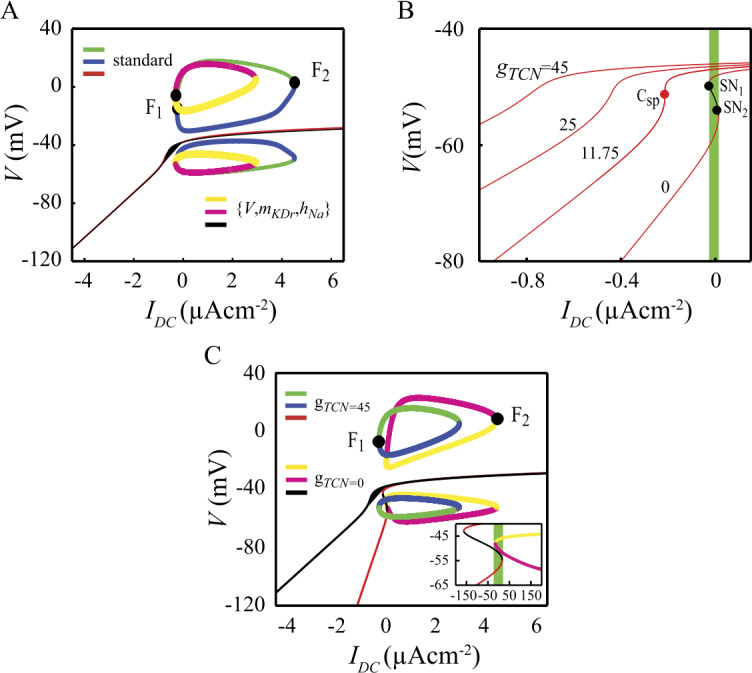
Properties of the $\{ V,m_{\text{Kdr}},h_{\text{NaV}} \} $ reduced model. A. Comparison of the bifurcation diagrams of the standard model and of the 3d reduced model. B. Effects of the $g_{\text{TCN}}$ conductance on the branch of fixed points in the bifurcation diagram of the 3d model. C. Effects of $g_{\text{TCN}}$ on the isola of limit cycles in the 3d model

We next investigated how the interactions between $I_{\mathrm{Na}}$, $I_{\mathrm{Kdr}}$ and $I_{\mathrm{TCN}}$ explain the coexistence of a branch of FP with an isola of LC in the DCNn. We have shown above that neither the Ca currents nor the Ca-dependent K currents are required to produce the basic bifurcation diagram of Fig. 2A. After withdrawing these currents, the standard model reduces to a simplified model with only three variables: the membrane potential *V*, the activation variable of $I_{\mathrm{Kdr}}$, $m_{\mathrm{Kdr}}$ and the inactivation variable of $I_{\mathrm{NaV}}$, $h_{\mathrm{NaV}}$. We therefore refer to this reduced model as to the $\{ V,m_{\mathrm{Kdr}},h_{\mathrm{NaV}} \} $ sub-system. Figure 7A shows that this sub-system retains the basic dynamical features of the standard model. In particular, the FP branch is nearly identical in the two models. Moreover, the $\{ V,m_{\mathrm{Kdr}},h_{\mathrm{NaV}} \} $ sub-system also exhibits an isola of LC albeit in a narrower range of tonic currents. Simulations of the reduced model reveal that pulses of injected current can trigger reversible $\mathrm{SD} \leftrightarrow \mathrm{F}$ like those described above in the full model (not illustrated). The FP branch exhibits a marked sensitivity to the magnitude of the $g_{TCN}$ conductance. Reducing this parameter from its standard value ($45~\mu \mbox{Scm}^{-2}$) shifts to the right the entire branch of FP along the $I_{\mathrm{DC}}$ axis. This result stems from the value of the $I_{\mathrm{TCN}}$ reversal potential ($E_{\mathrm{TCN}}=-34~\mbox{mV}$) that defines this current as inward (depolarizing) for all points of the FP branch, since they all are below $E_{\mathrm{TCN}}$. Hence, reducing $g_{\mathrm{TCN}}$ requires increasing the value of $I_{\mathrm{DC}}$ to achieve a FP with the same voltage. As $g_{\mathrm{TCN}}$ is reduced to $11.75~\mu \mbox{Scm}^{-2}$, the FP branch exhibits a point of infinite slope (red dot in Fig. 7B). This point corresponds to a cusp bifurcation. Below the cusp bifurcation, the FP branch is no longer stable for every $I_{\mathrm{DC}}$ value, but exhibits two saddle-node bifurcations ($SN_{1}$ and $SN_{2}$) that form a hysteresis loop (green rectangle in Fig. 7B) for the example of $g_{\mathrm{TCN}}=0$). Hence, the model has two coexisting stable silent modes of activity for values for $g_{\mathrm{TCN}}$ values below the cusp bifurcation. The isola of LC still exists in the $\{ V,m_{\mathrm{Kdr}},h_{\mathrm{NaV}} \} $ sub-system without $I_{\mathrm{TCN}}$ (Fig. 7C). But the model has now three stable attractors, the sLC and the two stable points of the FP branch mentioned above. Appropriate stimuli can theoretically trigger transitions between these three modes of activity in the range of $I_{\mathrm{DC}}$ values delimited by green box in the inset of Fig. 7C. These results therefore suggest that the functional role of $I_{\mathrm{TCN}}$ in the DCNn is to stabilize the SD state over the entire range of tonic currents provided by the synaptic inputs.

### Reduction of the model to 2D: evidence for a central role of $I_{\text{NaV}}$ in setting the DCNn electric personality

**Figure 8:**
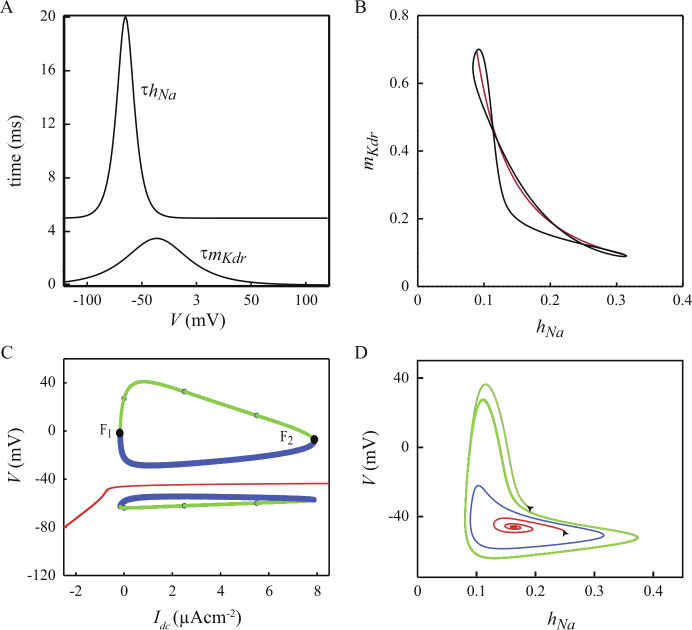
Reduction of the $\{ V,m_{\text{Kdr}},h_{\text{NaV}} \} $ sub-system to a 2d model. A. Time constants of the $m_{\text{Kdr}}$ and $h_{\text{NaV}}$ variables plotted as functions of the membrane potential *V*. B. Black: projection of the trajectory of a spike of the $\{ V,m_{\text{Kdr}},h_{\text{NaV}} \} $ sub-system onto the $\{ m_{\text{Kdr}},h_{\text{NaV}} \} $ subspace ($I_{\text{DC}}=0$). Red curve: best nonlinear fit of the mean spike trajectory (see text). C. Bifurcation diagram of the 2d reduced $\{ V,h_{\text{NaV}} \} $ model. D. Sample of characteristic trajectories of the 2d model in its phase space ($I_{\text{DC}}=0$). Blue: unstable LC. Green: trajectory of the 2d model starting from a state located outside the uLC orbit. Red: trajectory for initial conditions located inside the uLC orbit

The properties of the $\{ V,m_{\mathrm{Kdr}},h_{\mathrm{Na}} \} $ reduced model suggest that the interactions between $I_{\mathrm{NaV}}$ and $I_{\mathrm{Kdr}}$ play a crucial role in the emergence of the basic bifurcation diagram of the standard model. However, this sub-system does not allow a deep understanding of these interactions owing to their nonlinear nature. This prompted us to search for an even stronger reduction of the standard model to two dimensions, i.e. the minimum dimension for the emergence of limit cycles in ODE systems. To achieve this goal, we followed the approach of Fitzhugh [48] and searched for a relation between $m_{\mathrm{Kdr}}$ and $h_{\mathrm{NaV}}$ along the trajectory of a spike in the model ([48, 49] and see Rinzel [50] and Gerstner *et al*. [31] for developments of Fitzhugh’s mathematical approach on biophysical grounds). Fitzhugh observed that, in the squid axon, the projection of a spike trajectory in the $( m_{\mathrm{Kdr}}Oh_{\mathrm{NaV}} )$ plane is close to a straight line. This allowed one to lump together $m_{\mathrm{Kdr}}$ and $h_{\mathrm{Na}}$ into a so-called recovery variable, *w*, exploiting their linear relation interdependence. Combined with the assumption of instantaneous equilibrium for $m_{\mathrm{NaV}}$, this process allowed for the reduction of the original four-dimensional Hodgkin and Huxley model [16] to a simpler, two-dimensional model that keeps the fundamental dynamical features of the original model. Nevertheless, the implementation of this method of reduction of dimensionality also requires an appropriate expression for the (voltage-dependent) time constant $\tau _{w}$ for the new variable *w*. The solution was simple in the squid axon because the voltage dependencies of $\tau _{m_{\mathrm{Kdr}}}$ and $\tau _{h_{\mathrm{NaV}}}$ have similar shapes so that the voltage dependency of $\tau _{w}$ can be set as a trade-off between that of $\tau _{m_{\mathrm{Kdr}}}$ and $\tau _{h_{\mathrm{NaV}}}$ (see Fig. 17, Chap. 2 in [22]). On the opposite, in our DCNn model, the magnitudes and voltage-dependent characteristics of $\tau _{m_{\mathrm{Kdr}}}$ and $\tau _{h_{\mathrm{NaV}}}$ are too different to be reconciled by a single, trade-off behavior model for $\tau _{w}$ (Fig. 8A). We therefore turned to another approach and built 2d models that preserve either $\tau _{m_{\mathrm{Kdr}}}$ or $\tau _{h_{\mathrm{NaV}}}$. In our model the trajectory of a spike in the $\{ V,m_{\mathrm{Kdr}},h_{\mathrm{NaV}} \} $ model projected into the $( m_{\mathrm{Kdr}}Oh_{\mathrm{NaV}} )$ plane is not a simple curve (Fig. 8B). Like the projected curve of the Hodgkin–Huxley model, it exhibits two double points (see Fig. 6 in [49]) and we therefore searched for the best nonlinear fit of the trajectory in the $( m_{\mathrm{Kdr}}Oh_{\mathrm{NaV}} )$ plane. Firstly, we built a 2d model preserving the properties of $I_{\mathrm{Kdr}}$ by fitting the $h_{\mathrm{NaV}}$ versus $m_{\mathrm{Kdr}}$ relation. We found that the power law $h_{\mathrm{NaV}} = 0.07/m_{\mathrm{Kdr}}^{0.65}$ provides a good fit of the mean trajectory of a spike in the $( m_{\mathrm{Kdr}}Oh_{\mathrm{NaV}} )$ plane (not illustrated). By substituting this expression for $h_{\mathrm{NaV}}$ into the $I_{\mathrm{NaV}}$ equation we obtained a first 2d reduced model, the $\{ V,m_{\mathrm{Kdr}} \} $ sub-system, that comprises a unique voltage-dependent time constant $\tau _{m_{\mathrm{Kdr}}}$. However, this model fails to capture the features of the $\{ V,m_{\mathrm{Kdr}},h_{\mathrm{NaV}} \} $ model given that it fails to produce repetitive firing (not illustrated). We therefore turned to the alternative and fitted the $m_{Kdr}$ versus $h_{\mathrm{NaV}}$ relation. We found that this relation is well fitted by the equation $m_{\mathrm{Kdr}} = 0.0145/h_{\mathrm{NaV}}^{1.6}$. After substituting $m_{\mathrm{Kdr}}$ by this equation into the $I_{\mathrm{Kdr}}$ current equation, ones obtains a second 2d reduced model, the $\{ V,h_{\mathrm{NaV}} \} $ sub-system. Like the $\{ V,m_{\mathrm{Kdr}} \} $ sub-system, it contains a unique voltage-dependent time constant but it is $\tau _{h_{Na}}$ instead of $\tau _{m_{\mathrm{Kdr}}}$. Figure 8C shows that the $\{ V,h_{\mathrm{Na}} \} $ sub-system retains the qualitative dynamical features of the $\{ V,m_{\mathrm{Kdr}},h_{\mathrm{NaV}} \} $ model with an isola of LC coexisting with a branch of stable FP. The global properties of the $\{ V,h_{\mathrm{NaV}} \} $ sub-system can be understood geometrically since the model is 2d. Figure 8D depicts characteristic trajectories of the model for $I_{\mathrm{DC}}=0$. The blue closed curve is the trajectory of the uLC corresponding to $I_{\mathrm{DC}}=0$ in the bifurcation diagram. We found that any trajectory starting from an initial condition located inside the region bounded by the blue curve converges onto the FP point (see for example the red trajectory in Fig. 8D). On the opposite any trajectory starting outside the region defined by the blue curve converges onto the corresponding sLC in the bifurcation diagram (see for instance the green trajectory in Fig. 8D). We conclude that the blue curve is the separatrix between the attraction basins of the FP and of the sLC. Moreover, we observed that the $\{ V,h_{\mathrm{NaV}} \} $ sub-system loses the characteristic features of the $\{ V,m_{\mathrm{Kdr}},h_{\mathrm{NaV}} \} $ reduced model if $\tau _{m_{\mathrm{Kdr}}}$ rather than $\tau _{h_{\mathrm{NaV}}}$ is used in the $I_{\mathrm{NaV}}$ equation: the isola of LC disappears whereas the FP branch remains unaffected. This shows that the characteristics of the voltage-dependent time of inactivation of $I_{\mathrm{NaV}}$ are also fundamental for the coexistence of an isola of LC and of a branch of FP. Together these results suggest that the most important current for these dynamical features is $I_{\mathrm{NaV}}$.

### The triggering of reversible $\text{F} \leftrightarrow \text{SD}$ transitions by synaptic inputs

**Figure 9:**
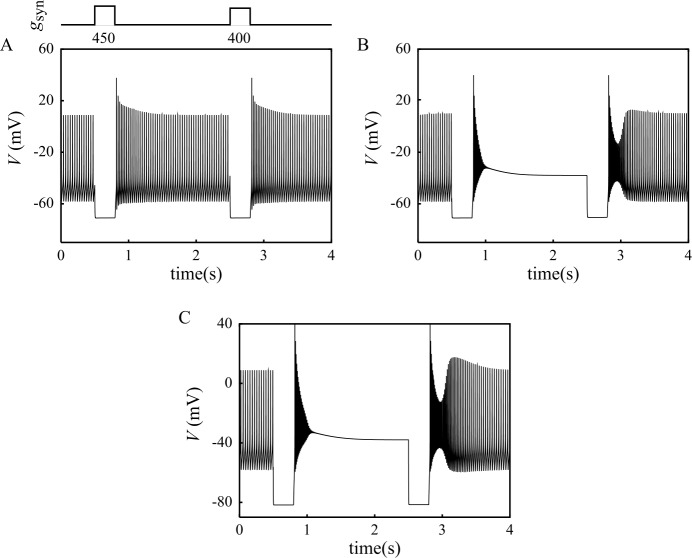
Triggering of $\text{F} \leftrightarrow \text{DB}$ transitions in the model by inhibitory (chloride) synaptic inputs. The traces illustrate the voltage response to square pulses (300 ms) of an inhibitory conductance. Labels indicate pulse magnitude in *μ*Scm^−2^. A. Standard model with a reversal potential $E_{\text{Cl}} = - 75~\mbox{mV}$. B. Variant model with voltage-dependent parameters of $I_{\text{CaT}}$ shifted to depolarized potentials by +10 mV (see text). C. Standard model with $E_{\text{Cl}} = - 85~\mbox{mV}$ (see text)

From a functional standpoint, it is crucial to determine whether $\mathrm{F} \leftrightarrow \mathrm{SD}$ transitions in DCNn actually occur in response to synaptic inputs provided by MF and PC. Indeed, the ideal phasic current sources used in previous sections have an infinite resistance whereas synaptic currents have a nonzero conductance that may prevent state transitions to occur by shunting the neuron membrane. Moreover, synaptic currents have an inversion potential which sets limits to voltage changes that can be triggered by activating these conductances. We therefore investigated this question with a variant model in which ideal phasic currents were replaced by models of synaptic currents, i.e. current sources having a nonzero conductance and an inversion potential: $I_{\mathrm{syn}} = g_{\mathrm{syn}} ( V - E_{\mathrm{syn}} )$. Experiments estimate the Nernst potential of Cl^−^ ions in DCNn to be $E_{\mathrm{Cl}} = - 75~\mbox{mV}$ [3, 51]. Figure 9A illustrates the response of the standard model to a pair of successive pulses of synaptic current that model the inhibitory inputs of PC synapses onto the DCNn. Both pulses (of different amplitudes) interrupt the tonic firing of spikes in the model in agreement with experimental observations that PC inputs can silence DCNn [51]. After the pulse, the DCNn, however, resumes firing spikes after a ${\sim} 300~\mbox{ms}$ period of increased firing frequency. We found that such synaptic inhibitory pulses cannot trigger a $\mathrm{F} \to \mathrm{DB}$ transition in those conditions, whatever the duration or amplitude of the pulse (data not shown). Examination of $I_{\mathrm{CaT}}$ dynamics during the pulse showed that inhibitory synaptic conductances de-inactivate a smaller amount of $I_{\mathrm{CaT}}$ compared to direct current injections, thus explaining the absence of $\mathrm{F} \to \mathrm{SD}$ transition by inhibitory synaptic conductances. These results may lead one to conclude that physiological inputs from PC are unable to trigger $\mathrm{F} \to \mathrm{SD}$ transitions in DCNn. However, Boehme *et al*. [52] have observed that large synaptic inhibition of DCNn by Purkinje cells potently elicit rebound potentials that are underlain by $I_{\mathrm{CaT}}$. These authors suggested that these observations could result from an erroneous estimate of either the voltage dependence of $I_{\mathrm{CaT}}$ or the value of the chloride Nernst potential $E_{\mathrm{Cl}}$. They noticed that the previous estimates of the voltage characteristics of $I_{\mathrm{CaT}}$ have been derived from DCNn recordings made in the soma where the density of $I_{\mathrm{CaT}}$ is smaller than in the DCNn dendrites [14]. Owing to difficulties to achieve perfect space-clamp of the membrane voltage over the entire neuron surface, these authors estimated that the actual voltage-dependent parameters of $I_{\mathrm{CaT}}$ could be less negative by as much as 10 mV. Figure 9B illustrates the model response to the same stimulation protocol as Fig. 9A but after a +10 mV shift of the voltage-dependence of $I_{\mathrm{CaT}}$. The first current pulse now is able to trigger a $\mathrm{F} \to \mathrm{SD}$ transition while the second pulse induces the opposite transition, switching back the model to its F state. Boehme *et al*. [52] further suggested that $E_{\mathrm{Cl}}$ in the DCNn may also be wrongly estimated by whole cell recordings since the patch pipette imposes its electrolytes composition to the recorded neuron. They estimated that the physiological $E_{\mathrm{Cl}}$ could be more negative, down to −85 mV. This negative shift of $E_{\mathrm{Cl}}$ induces effects that are qualitatively identical to positive shifts of the $I_{\mathrm{CaT}}$ voltage-dependency (Fig. 9C), the first pulse triggering a $\mathrm{F} \to \mathrm{SD}$ transition and the second pulse resetting the neuron to its firing state.

## Discussion

The mathematical model presented in this study sketches an original electric personality for the neurons of the deep cerebellar nuclei (DCNn) which remained unsuspected until now in spite of experimental data evidencing puzzling state transitions in these neurons. The pioneer investigation of DCNn intrinsic electric properties by Jahnsen ([2] and [51]) reported that spontaneously active DCNn can be switched to a silent state and back to their firing mode by pulses of injected current (Fig. 5 in [2]). Jahnsen referred to the silent state as a ‘steady depolarization and inactivation of the spikes’ and our terming ‘SD sate’ stands as a shortcut for this description. The study of Raman *et al*. [4] subsequently reported strikingly similar results with patch-clamp recordings showing that depolarizing current pulses can switch DCNn up to a silent depolarized state until the membrane is actively hyperpolarized by a current injection. Notice that the calling of this state a ‘depolarization block’ (DB) by Raman *et al*. [4] actually appears misleading. Indeed, many (if not all) neurons exhibit an inactivation of their spike mechanism in response to large depolarizing currents which is called DB (see e.g. midbrain dopamine neurons [53], hippocampal CA1 pyramidal neurons [54] and layer 5 pyramidal neuron of rat’s visual cortex [43]). However, the DB does not outlast the duration of its triggering depolarizing current in all cases that we aware of, showing that the DB is not a self-sustained state. In order to highlight these differences, we have chosen to term lasting depolarizations of DCNn as a ‘SD state’, this acronym standing for a shortcut of properties of these lasting sustained depolarizations initially described by Jahnsen [2]. Experimental data of Jahnsen [2] and Raman *et al*. [4] therefore suggest that the SD state may be a genuine state of the DCNn distinct from their spontaneously firing regime (F mode). Since a previous model of DCNn intrinsic electrical properties did not reproduce the coexistence of distinct firing regimes [7], we have built a new model to investigate the hypothesis of a stable depolarizing state in the DCNn, its relation with the spontaneous firing state and interactions between membrane ion currents that give rise to the coexistence of these two states.

Our model uses the latest experimental findings on active membrane ion currents in DCNn to suggest that DCNn have two coexisting stable electrical states. One them is the F mode in which neurons fire fast spikes at low spontaneous frequencies in agreement with *in vitro* studies [2–7]. The other state is a silent mode of activity characterized by a depolarized membrane potential at around −38 mV. We call it the SD state (for stable depolarized state) to stress the proposition that it is locally stable and excitable according to our model. This is evidenced by the fact that our model can be switched back and forth between its SD and F modes by brief pulses of de- or hyperpolarizing currents (see Fig. 1). According to our results (Fig. 2), these bistability properties of the DCNn may reflect the fact that these neurons lay at rest between two-fold bifurcations delimiting an isola of limit cycles which coexists with a branch of stable fixed points. The FP for $I_{\mathrm{DC}}=0$ would correspond to the plateau depolarization reported by Jahnsen [2] and by Raman *et al*. [4]. To our knowledge, such dynamical properties have been previously described only in the squid axon bathed in low $[ \mathrm{Ca} ]_{0}$ saline [12].

Our model is a single-compartment, isopotential simplification of a cell that actually exhibits a more complex morphology with dendrites and soma. This simplification is in part justified by previous modeling studies of the passive properties of these cells that suggest that these cells are moderately electrotonically compact [7]. A first extension of our model consists in lumping the whole dendritic tree into a unique effective isopotential compartment and connecting it to our initial soma model with a coupling conductance. Preliminary investigation of the resulting two-compartment system suggests that the capacitive and passive resistance loads imposed by the dendritic compartment to the soma are unlikely to challenge the basic mechanism that we propose to explain the transitions from the firing to the SD state in DCNn. In fact, the documented enrichment of DCNn dendrites in CaT current (14) could even boost the facility with which hyperpolarizing inputs can trigger these transitions.

### Experimental testing of the model predictions

Our analysis of the mechanisms underlying $\mathrm{F} \leftrightarrow \mathrm{SD}$ transitions with the standard version of the model and variant versions mimicking pharmacological block of the different membrane ion currents suggests three experiments to unambiguously test the hypothesis of F–SD states bistability in the DCNn. These experiments could be achieved with standard intracellular recording of DCNn in cerebellum slices. *Experiment I—evidence for a branch of SD states*: our model predicts that recorded DCNn should reach a silent state of activity upon injecting them with sufficiently large amounts of steady depolarizing currents. Notice that this behavior is expected for any neuron type whose rising phase of the spike is underlain by a voltage-dependent Na current exhibiting inactivation. Unlike other neurons however, our model predicts that a DCNn should remain in this silent state when the amplitude of injected currents is decreased back at a rate sufficiently slow to prevent crossing of the separatrix between the F and SD states. Then, starting from hyperpolarized membrane potentials, our model predicts that the recorded DCNn should remain silent when the magnitude of the hyperpolarizing current is slowly reduced down to zero and even upon injecting large depolarizing currents. *Experiment II—evidence for an isola of limit cycles*: this second experiment investigates the firing mode of the DCNn with the help of standard (firing frequency vs $I_{\mathrm{DC}}$) curves. Injecting depolarizing steady currents of growing magnitude into spontaneously active DCNn should increase their firing frequency up to a maximum current value ($I_{F2}$ in Fig. 1) beyond which the DCNn should switch to a silent state. Conversely, in a spontaneously firing DCNn, increasing the magnitude of the tonic hyperpolarizing current above $I_{F1}$ should abruptly switch the neuron to its silent mode. The observation of this result would lead one to categorize the DCNn as type II neurons according to the Hodgkin classification [42]. The latter part of this experiment should especially be achieved in the presence of broad-spectrum inhibitors of postsynaptic channels to remove spontaneous synaptic activity in slices. Membrane potential fluctuations due to ongoing synaptic activity would hinder determining if the DCNn minimal firing frequency is actually nonzero. *Experiment III—changing the external* Ca^2+^ *concentration*: this experiment tests the model prediction that the DCNn can be made to adopt the classical bifurcation scenario for type II neurons, by turning the left-hand side fold bifurcation of the isola into a subcritical Hopf bifurcation and the right one into a supercritical Hopf bifurcation (see [43]) by increasing $[ \mathrm{Ca} ]_{0}$. These changes of bifurcation would not be accompanied by qualitative modification of the f/I relationship. But they could be identified by a continuous decrease of the spike amplitude down to zero upon increasing the driving current up to the right-hand side bifurcation. *Experiment IV—extent of* $I_{\mathrm{Na}}$ *inactivation in the SD state*: intracellular recordings could (i) check that $I_{\mathrm{Na}}$ is not fully inactivated in the SD state and (ii) investigate whether the full blockage of $I_{\mathrm{Na}}$ with TTX prevents DCNn state transitions by removing the SD state.

### Ionic currents and dynamics of the model

**Figure 10:**
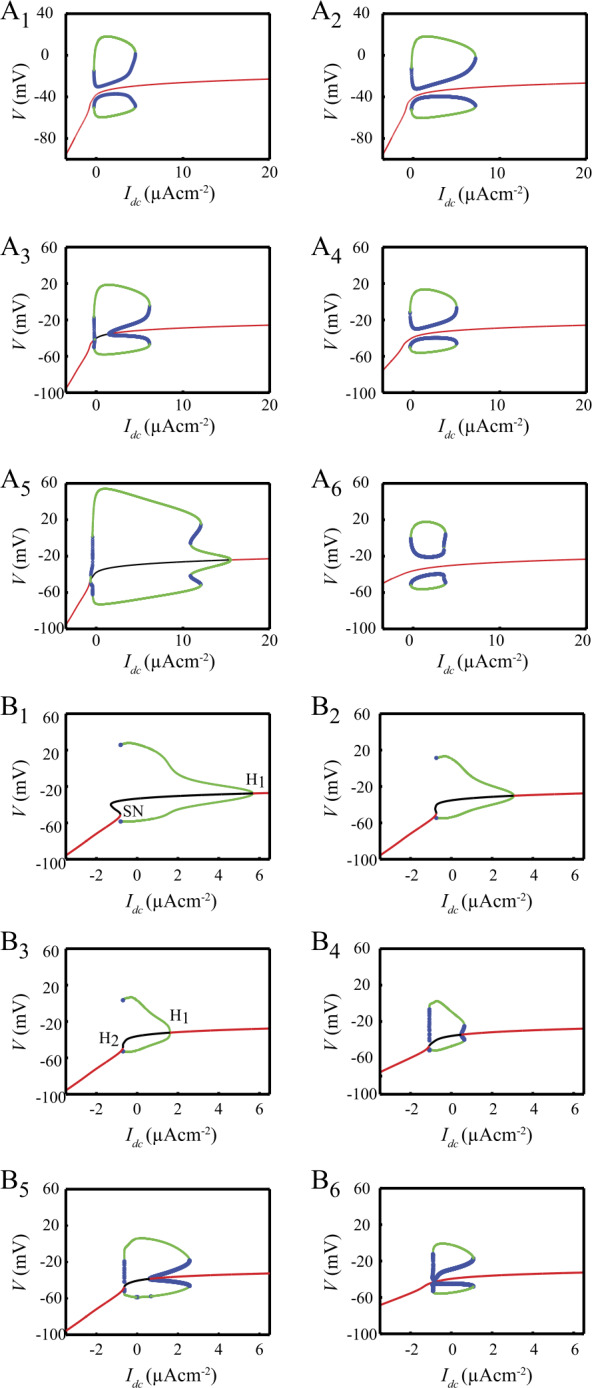
Robustness of the model’s bifurcation diagram to parameters-models of $I_{\text{Kdr}}$ and $I_{\text{NaV}}$ (see text). A. Impact of putative Kv2 channels. Symbol $I_{\text{KdrS}}$ stand for the standard $I_{\text{Kdr}}$ after its voltage-dependent time constant was scaled up to model (slow) Kv2 channels. A_1_. Standard model. A_2_. After addition of $I_{\text{KdrS}}$ (with the same conductance as $I_{\text{Kdr}}$). A_3_. Same as A_2_ with $g_{\text{Na}}$ decreased to $3000~\mu \mbox{Scm}^{-2}$. A_4_. Same as A_3_ with $g_{\text{TCN}}$ increased to 75*μ*Scm^−2^. A_5_. With $I_{\text{KdrS}}$ as the sole voltage-dependent K current (mode’s standard conductance value). A_6_. Same as A_5_ with $g_{\text{TCN}}=215~\mu \mbox{Scm}^{-2}$. B. Impact of the persistent component of $I_{\text{NaV}}$ identified in DCNn. Symbol $I_{\text{NaRB}}$ stands for the model of voltage-dependent Na current of Raman and Bean [55]. B_1_. $I_{\text{NaV}}$ replaced by $I_{\text{NaRB}}$ with conductance $g_{\text{NaRB}} = 5 \times 10^{3}$ (same as that of $I_{\text{NaV}}$ in the standard model). B_2_. $g_{\text{NaRB}} = 3.5 \times 10^{3}$. B_3_. $g_{\text{NaRB}} = 3 \times 10^{3}$. B_4_. Same as B_3_ with $g_{\text{TCN}} = 75$ (standard value = 45). B_5_. With $g_{\text{Kdr}} = 1.25 \times 10^{4}$ (standard value = 4.5 × 103). B_6_. $g_{\text{NaRB}} = 3 \times 10^{3}$, $g_{\text{Kdr}} = 1.25 \times 10^{4}$ and $g_{\text{TCN}} = 9.5 \times 10^{1}$

The analysis of the standard model and its lower dimension variants suggest that the voltage-dependent properties of Na currents are of paramount importance in the state transitions displayed by the DCNn *in vitro*. In order to justify this major conclusion, we come back to the biophysical foundations of our model, namely the set of ion currents extracted from the experimental literature on the DCNn to build our model and their mathematical formulation. To avoid introducing unjustified hypotheses, we assumed that voltage-dependent currents obey the classical Hodgkin–Huxley formalism which has proven capable to reproduce the qualitative dynamics of most neuron types studied so far with mere parameter adjustments. For $I_{\mathrm{Kdr}}$ this hypothesis is consistent with the finding by Raman *et al*. [4] of a classical delayed rectifier K current in DCNn. However, the large time constant of activation reported in this study (12 ms at +12 mV) suggests that this voltage-dependent current is carried by (slow) Kv2 channels (see e.g. [29]) whereas other studies give no evidence for the expression of Kv2 channels by DCNn. These studies rather show that DCNn express (fast) Kv3 channels and that these channels are functional (see Methods). Our model was accordingly designed with a voltage-dependent time constant for $I_{Kdr}$ corresponding to fast Kv channels. Nevertheless, we investigated the possible involvement of Kv2 channels in the DCNn electro-responsiveness with a variant model comprising a slowly activating K current, $I_{\mathrm{KdrS}}$, in addition to $I_{\mathrm{Kdr}}$. $I_{\mathrm{KdrS}}$ was given the same voltage-dependence as $I_{\mathrm{Kdr}}$ but its time constant was multiplied by 11 to achieve a value of 12 ms at +12 mV. Adding $I_{\mathrm{KdrS}}$ to the model with the same conductance as $I_{\mathrm{Kdr}}$ did not change the overall structure of the model’s bifurcation diagram (compare panels A and B in Fig. 10). However, it increased the width of the isola of LCs and reduced the spontaneous firing frequency from 28.9 Hz to 25.5 Hz. This frequency could even be reduced to 20 Hz (the spontaneous firing frequency of DCNn *in vitro* [2–7]) by increasing the $g_{KdrS}$ value to 3.3 times that of $g_{\mathrm{Kdr}}$ (not illustrated). These results suggested that previous studies of Kv channels in DCNn may have missed the Kv2 channels implied by the results of Raman et al. [4] and that currents through Kv2 channels underlie the low spontaneous frequency firing of DCNn. To address this hypothesis, we fixed the value of $g_{\mathrm{KdrS}}$ and decreased that of $g_{\mathrm{Kdr}}$ to determine to what extent slow $\mathrm{K}_{\mathrm{V}}3$ channels are mandatory to explain the observed firing dynamics of DCNn. Decreasing $g_{\mathrm{Kdr}}$ diminishes the width of the isola of LC down to $g_{\mathrm{Kdr}}=3 \times 10^{3}~\mu \mbox{Scm}^{-2}$, where the branch of SD states loses its stability (Fig. 10C check). Nevertheless, overall stability of the FP branch was restored by increasing the conductance of $I_{\mathrm{TCN}}$ to $3 \times 10^{3}~\mu \mbox{Scm}^{-2}$, suggesting that the functional role of $I_{\mathrm{TCN}}$ is to stabilize the SD state of DCNn. We tested this proposal by reducing $g_{Kdr}$ down to the complete withdrawal of the fast $\mathrm{K}_{\mathrm{V}}$ current in the model. The range of tonic currents over which the FP branch is unstable in the hybrid Kdrs–Kdr variant model (Fig. 10B) was even more enlarged after switching the $I_{\mathrm{Kdr}}$ model to solve Kv2-like dynamics (Fig. 10E). Nevertheless, increasing $g_{\mathrm{TCN}}$ proved again capable to resolve this issue as shown by panel F in Fig. 10 displaying the bifurcation diagram of the model endowed with $g_{\mathrm{TCN}}$ to $215~\mu \mbox{Scm}^{-2}$. Beyong strengthening our proposal on the functional role of ITCN, these results demonstrate that the basic bifurcation scenario of the standard model can be achieved by a model comprising only slow Kv channels.

Adequacy of the classical HH formalism to model voltage-dependent Na currents in DCNn proved more challenging owing to two reasons: (i) the integer power to which raise variable $m_{\mathrm{NaV}}$ and (ii) the evidence for a resurgent Na current in DCNn (Ref) not introduced in our model. Raman *et al*. [4] fitted their data for the steady-state activation of Na currents with a Boltzmann function of the form given by Eq. (5) for $m_{\mathrm{NaV}\infty } $ (see their Fig. 3C). The standard HH formalism for this current uses the same function but raised to a power of three (see e.g. [26]), i.e. $m_{\mathrm{NaV}\infty }^{3}$. Assuming that the error between the data points and the model prediction are independently and identically distributed according to a normal law, we computed the Bayesian information criterion (BIC) for both models [56]. We found that $\mathrm{BIC}_{m_{\infty }} - \mathrm{BIC}_{m_{\infty }^{3}} > 16$, which means that the standard $m_{\mathrm{NaV}\infty }^{3}$ formalism yields a better description of the experimental data than a $m_{\mathrm{Na}\infty } $ formalism. The classical HH formalism hence appears to be capable to describe adequately the transient component of $I_{\mathrm{NaV}}$ currents in DCNn. Notice that the two studies on $I_{\mathrm{NaV}}$ in DCNn that we are aware of ([4] and [18]) provide divergent results regarding the inactivation parameters of this current. Our model adopts as standard parameters the values reported in [18] in physiological salines rather than values in [4] as the latter were obtained in low external Na conditions. Nevertheless, we found that the basic bifurcation diagram and $\mathrm{F} \leftrightarrow \mathrm{SD}$ state transitions are robust to changes in the inactivation parameters of $I_{\mathrm{NaV}}$ which include the values reported in [4], thereby showing that these properties do not result from an unrealistically precise setting of the $I_{\mathrm{NaV}}$ parameters. We then investigated the possible role of the resurgent component of $I_{\mathrm{NaV}}$ reported in DCNn by Afshari *et al*. [18]. This current, which was initially identified in cerebellar Purkinje cells [55], corresponds to a transient block of NaV channels in their open state that is not accounted for by the classical HH formalism. For this reason, we investigated the properties of a variant model in which $I_{\mathrm{NaV}}$ was modeled with the state transition scheme of Raman and Bean [19], which produces a resurgent current. Figure 10B summarizes the results of this investigation. When endowed with $I_{\mathrm{NaRB}}$ instead of the standard $I_{\mathrm{NaV}}$ (same conductance as $I_{NaV}$), the model can no longer produce $\mathrm{F} \leftrightarrow \mathrm{SD}$ transitions due to the loss of overall stability of the FP branch (Fig. 10B_1_). The exchange of Na current models also induces the appearance of saddle-node (SN) bifurcation in this branch. Rather than forming an isola of LC as in the standard model, the branch of sLC ends into a homoclinic bifurcation at regular saddle at the left of the bifurcation diagram and into a Hopf bifurcation ($H_{1}$) at the right of the diagram. As $I_{\mathrm{NaRB}}$ adds a resurgent component to the transient Na current modeled with $I_{\mathrm{NaV}}$, we reasoned that loss of stability of the FP may stem, at least partly, from the Na current having become excessively large. In agreement with this reasoning, panels B_2_ and B_3_ in Fig. 10 show that a progressive decrease of $g_{\mathrm{NaRB}}$ shifts the $H_{1}$ point to the left of the bifurcation diagram. It also removes the SN bifurcation point to leave a second Hopf bifurcation point ($H_{2}$) which shifts to the right of the bifurcation diagram as $g_{\mathrm{NaRB}}$ is decreased furthermore. However, decreasing $g_{\mathrm{NaRB}}$ alone proved unable to restore the standard bifurcation diagram as the branch of LCs was lost when the FP resumed overall stability (not illustrated). As our study suggests that the $I_{\mathrm{TCN}}$ current contributes to the stability of the FP branch, we then increased $g_{\mathrm{TCN}}$ in addition to decreasing $g_{\mathrm{NaRB}}$. Figure 10B_4_ shows this additional parameter change brought the $H_{1}$ and $H_{2}$ closer to each other and allowed the model to recover two short branches of uLC at ends of the branch of sLC. However, further increases in $g_{\mathrm{TCN}}$ proved unable for the variant model to recover the isola of LC as they exchanged the stability of the left uLC branch (not illustrated). Given that $I_{\mathrm{Kdr}}$ exerts a counteracting effect to $I_{\mathrm{Na}}$, we finally examined the effects of increasing $g_{\mathrm{Kdr}}$. Figure 10B_5_ shows that increasing $g_{\mathrm{Kdr}}$ has similar effects to that of increasing $g_{\mathrm{TCN}}$. However, increasing $g_{\mathrm{Kdr}}$ alone also proved unable for the model to recover the isola of LC (not illustrated). Nevertheless, panel B_6_ shows that combining the three effects of decreasing $g_{\mathrm{NaRB}}$ and increasing $g_{\mathrm{TCN}}$ and $g_{\mathrm{Kdr}}$ allows the variant model to recover the overall structure of the bifurcation diagram of the standard model. These results show that a variant model endowed with an accurate biophysical model of $I_{\mathrm{NaV}}$ including a resurgent component can produce a bifurcation diagram consistent with that of the standard model and therefore support the mechanism that our study proposes to explain $\mathrm{F} \leftrightarrow \mathrm{SD}$ transitions of DCNn.

Given that not all DCNn currents have been thoroughly characterized, matching DCNn salient electrophysiological properties (spontaneous spiking frequency, *f*–*I* relationship, spikes under- and overshoot values and voltage of the SD state) resulted in trade-off values for parameters of $I_{\mathrm{NaV}}$ (i.e. $V_{m_{\mathrm{NaV}}}$, $k_{m_{\mathrm{NaV}}}$, $V_{h_{\mathrm{NaV}}}$, and $k_{h_{\mathrm{NaV}}}$) in our model that deviate from mean experimental values documented in Raman *et al*. [4] and Afhsari *et al*. [18]. Nevertheless, we observed that dynamical properties of our model, including its $\mathrm{F} \leftrightarrow \mathrm{SD}$ states transitions, could be retained when using these mean experimental values provided that membrane conductances $g_{\mathrm{NaV}}$ and $g_{\mathrm{TCN}}$ were adjusted in ways that strictly agree with our findings on the effects of these conductances on the bifurcation diagram of the model.

At least two different kinds of DCNn have been characterized: GABAergic DCNn and non-GABAergic DCNn [24]. The former exhibit a lower spontaneous firing frequency (∼10 vs ∼30 Hz) and a lower maximal firing frequency than the latter (∼50 vs >100 Hz). With standard parameter values, our model has a spontaneous firing frequency of ∼29 Hz and a maximal frequency of ∼110 Hz, so our model unambiguously corresponds to non-GABAergic large neurons. From a preliminary exploration of the parameter space, we think that it may be possible to reproduce the firing range of GABAergic DCNn with our model. The spontaneous firing rate mostly depends on the TCN current, so decreasing this conductance leads to a decreased spontaneous firing rate. However, this also alters the bifurcation diagram, destroying the isola of limit cycles and suppressing the possibility of transitions between a firing and a SD state. However, it is not clear if those transitions do arise in GABAergic DCN experimentally. Therefore, our model with its standard parameter values corresponds to non-GABAergic DCNn and further work is needed to determine if it can be adapted to account for GABAergic DCN too.

### Functional implications

An unresolved central issue in the cerebellum functioning is the observation that activities of connected Purkinje cells (PC) and DCNn can exhibit negative as well as positive correlations [57]. The observation of a positive correlation is puzzling since PC inhibit DCNn (with a ratio of 800 PCs to 1 DCNn) so that one would expect negative correlations between PC and DCNn firings. The basic bifurcation scenario of our DCNn model offers a fresh look at this question. It suggests that salient sensorimotor information transmitted by the phasic inhibitory inputs of Purkinje cells or the excitatory inputs of mossy fibers collaterals can trigger reversible ‘on’–‘off’ transitions of the DCNn (see Fig. 6). According to our model a DCNn can be in its firing ‘on’ state even if PC targeting it are active provided that the total tonic inhibitory current fed by PC is not large enough to shift the model below the left bound of the isola of limit cycles (see Fig. 2A). Moreover, our model predicts that the state of an initially silent DCNn after a PC inhibitory signal depends on the amplitude of the input: moderate-amplitude inputs will switch the DCNn to its firing state whereas large-amplitude inputs will keep the DCNn in the silent state. Because of this counter-intuitive behavior, the activity of the DCNn and of the PC can exhibit positive and negative correlations, as reported by McDevitt *et al*. [57]. Our findings therefore strongly suggest to study computational models of the cerebellum network endowed with the basic DCNn state transition features disclosed by our study.

## Figures and Tables

**Figure 11:**
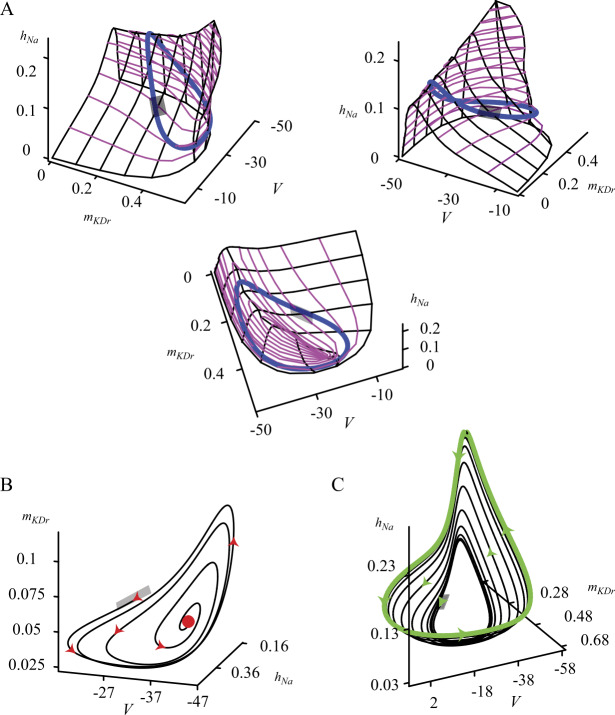
A. Sampling of the separatrix between the basins of attraction of the SD and the sLC for three different orientations. Curves tangent to the separatrix were computed for different fixed values of one the model state variables. Red: $m_{\text{Kdr}}=0.3$, green: $h_{\text{NaV}}=0.06$, Blue: $m_{\text{Kdr}}=0$, magenta: $h_{\text{NaV}}=0$. Black curve: uLC. Gray box: flat surface patch tangent to the separatrix; light and dark grays, respectively, indicates inside and outside of the separatrix surface. B. The model converges to its SD state from a state point of the phase space located inside the phase space volume bounded by the separatrix. C. Convergence of the model to the sLC from initial conditions located outside of this volume. All data depicted in the figure were computed with $I_{\text{DC}} = 0$

## Data Availability

Source codes for numerical simulations available upon request to the authors.

## Appendix: On the separatrix between the basins of attraction of the SD and F states

We show in the Methods section that solutions of our model are bounded. Moreover, our model has no strange (fractal dimension) attractor in addition to the F and SD attractors. It follows that any trajectory of the model, whatever its initial conditions, must eventually converge on either the F or the SD attractor. This implies the existence of a closed manifold (i.e. compact and without bounds) separating the phase space. This leads us to propose that the separatrix delimiting the basins of attraction of the F and SD attractors is a $(n-1)$-dimension closed manifold. We give here information supporting this hypothesis.

A virtue of the reduced $\{ V,m_{\mathrm{Kdr}},h_{\mathrm{Na}} \} $ model is that it possesses a three-dimensional phase space allowing one to visualize its dynamics, contrary to the standard six-dimensional system. We used this property to study in more detail the separatrix between the basins of attraction of the SD and F states. We were able to sample the separatrix in the $\{ V,m_{\mathrm{Kdr}},h_{\mathrm{NaV}} \} $ model by imposing a fixed value to two its three state variables model and then finding by dichotomy values of the remaining variable that are closest to the separatrix. We illustrate the results in Fig. 11A for three different orientations in the $\{ V,m_{\mathrm{Kdr}},h_{\mathrm{NaV}} \} $ phase space. The black mesh locates the points of the separatrix sampled for different fixed values of either $m_{\mathrm{Kdr}}$ or $h_{\mathrm{NaV}}$. This mesh clearly forms a closed surface showing that the separatrix in the $\{ V,m_{\mathrm{Kdr}},h_{\mathrm{NaV}} \} $ reduced model is two-dimensional. The small parallelogram in panels A represents a flat surface locally tangent to the separatrix at a point in the phase space with coordinates ($V = - 30.1$, $m_{\mathrm{Kdr}} = 0.3$, $h_{\mathrm{NaV}} = 0.06 $). The inner side of this surface is depicted in light gray and its outer side in dark gray. Figure 11B illustrates an example of the fate of all trajectories starting from initial conditions located inside the volume delimited by the separatrix: all these trajectories eventually converge to the SD state (red dot). On the opposite, all trajectories of the model starting from a point located outside of this volume converge onto the sLC (green curve) as illustrated by Fig. 11C. These results confirm that the surface delineated by the black mesh in Fig. 11A$_{1\text{--}3}$ is the separatrix between the attraction basins of the F and SD states. Numerical investigation of the uLC in the $\{ V,m_{\mathrm{Kdr}},h_{\mathrm{NaV}} \} $ reduced model revealed that this limit cycle has one of its Floquet exponents equal to 1 (as expected from Floquet theory, (see e.g. [13])), a second one being <1 and the remaining one being >1. Hence, the local unstable manifold of the uLC is of dimension 1 whereas its local stable manifold is of dimension 2. Since the global stable and unstable manifolds of the uLC are locally tangent, respectively, to the local stable and unstable manifolds in a neighborhood of the uLC and according to the above results showing that the separatrix is a closed surface, we are led to propose that the SD/F separatrix is actually the global stable manifold of the uLC. The finding that this manifold is of dimension 2 agrees with the hypothesis that the separatrix is of dimension $n-1$ in the $\{ V,m_{\mathrm{Kdr}},h_{\mathrm{NaV}} \} $ reduced model. Moreover, numerical analysis of the uLC in the standard six-dimensional model revealed that one of its Floquet exponents is equal to 1, four of them are <1 and the last one >1 so that the sLC stable manifold is of dimension 5. According to the conclusion derived from the $\{ V,m_{\mathrm{Kdr}},h_{\mathrm{NaV}} \} $ reduced model, it follows that the F/SD separatrix is likely of dimension 5 in the standard model, thereby providing support for the $n - 1$ dimension hypothesis on the separatrix.

## Abbreviations

DCNn: neurons of the deep cerebellar nuclei
F state: firing of spikes state
SD state: stable depolarized state
PC: Purkinje cells
sFP: stable fixed point
sLC: stable limit cycle
uLC: unstable limit cycle
ODE: ordinary differential equations
